# Longitudinal Multi-Omics Study of a Mother-Infant Dyad from Breastfeeding to Weaning: An Individualized Approach to Understand the Interactions Among Diet, Fecal Metabolome and Microbiota Composition

**DOI:** 10.3389/fmolb.2021.688440

**Published:** 2021-10-04

**Authors:** Giorgia Conta, Federica Del Chierico, Sofia Reddel, Federico Marini, Fabio Sciubba, Giorgio Capuani, Alberta Tomassini, Maria Enrica Di Cocco, Nicola Laforgia, Maria Elisabetta Baldassarre, Lorenza Putignani, Alfredo Miccheli

**Affiliations:** ^1^ Department of Chemistry, Sapienza University of Rome, Rome, Italy; ^2^ NMR-Based Metabolomics Laboratory of Sapienza (NMLab), Sapienza University of Rome, Rome, Italy; ^3^ Multimodal Laboratory Medicine Research Area, Unit of Human Microbiome, Bambino Gesù Children’s Hospital, IRCCS, Rome, Italy; ^4^ Department of Biomedical Science and Human Oncology, University of Bari Aldo Moro, Bari, Italy; ^5^ Department of Diagnostic and Laboratory Medicine, Unit of Microbiology and Diagnostic Immunology, Unit of Microbiomics and Multimodal Laboratory Medicine Research Area, Unit of Human Microbiome, Bambino Gesù Children’s Hospital, IRCCS, Rome, Italy; ^6^ Department of Environmental Biology, Sapienza University of Rome, Rome, Italy

**Keywords:** gut microbiota, NMR-based metabolomics, metabolic profiling, breast milk, targeted metagenomics, HMOs, omics data integration

## Abstract

The development of the human gut microbiota is characterized by a dynamic sequence of events from birth to adulthood, which make the gut microbiota unique for everyone. Its composition and metabolism may play a critical role in the intestinal homeostasis and health. We propose a study on a single mother-infant dyad to follow the dynamics of an infant fecal microbiota and metabolome changes in relation to breast milk composition during the lactation period and evaluate the changes induced by introduction of complementary food during the weaning period. Nuclear Magnetic Resonance (NMR)-based metabolomics was performed on breast milk and, together with 16S RNA targeted-metagenomics analysis, also on infant stool samples of a mother-infant dyad collected over a period running from the exclusive breastfeeding diet to weaning. Breast milk samples and neonatal stool samples were collected from the 4th to the 10th month of life. Both specimens were collected from day 103 to day 175, while from day 219–268 only stool samples were examined. An exploratory and a predictive analysis were carried out by means of Common component and specific weight analysis and multi-block partial least squares discriminant analysis, respectively. Stools collected during breastfeeding and during a mixed fruit/breastfeeding diet were characterized by high levels of fucosyl-oligosaccharides and glycolysis intermediates, including succinate and formate. The transition to a semi-solid food diet was characterized by several changes in fecal parameters: increase in short-chain fatty acids (SCFAs) levels, including acetate, propionate and butyrate, dissapearance of HMOs and the shift in the community composition, mainly occurring within the *Firmicutes* phylum. The variations in the fecal metabolome reflected the infant’s diet transition, while the composition of the microbiota followed a more complex and still unstable behavior.

## 1 Introduction

The human body can be considered as a holobiont, namely the complex ecosystem that involves not only the interrelations across the activities of different cells, tissues and organs, but also those with the microbiota colonizing the host (van de Guchte et al., 2018). The gut microbiota plays a major role in health and disease in humans by transforming the dietary compounds that are not directly digestible by the host enzymes, as well as by providing intestinal protection from pathogen colonization, inducing cell differentiation and modulation of the immune system of the host (Lynch and Pedersen, 2016; Mulligan and Friedman, 2017). The gut microbiota is unique to each individual and originates during childbirth, even though recent studies have provided evidence for the presence of bacteria in the fetal gut prior to birth, meaning that colonization could occur prenatally (Blaser and Dominguez-Bello, 2016; Walker et al., 2017). The microbial colonization of a healthy human gut is characterized by a dynamic sequence of events from birth to adulthood, playing a pivotal role in promoting intestinal homeostasis. Its composition varies throughout life (Yatsunenko et al., 2012). Type of delivery, pregnancy complications, preterm birth, antibiotic exposure, environmental factors including geographical background and household exposures, use of complementary formula milk and age at the start of weaning are all well-known factors that act on the inter-individual variability in the first year of life (Claesson et al., 2011; Lozupone et al., 2012; Wampach et al., 2017; Iozzo and Sanguinetti, 2018; Fettweis et al., 2019; Bayar et al., 2020; Differding et al., 2020). Gut microbiota can affect the host metabolism via processes including energy harvesting from diet, modulation of lipid metabolism, endocrine function and inflammatory response (Nyangale et al., 2012; Nauta et al., 2013), hence its compositional alterations, especially during early life, may lead to pediatric disorders and/or, later in life, contribute to the onset of diseases (Milani et al., 2017). The development of gut microbiota during breastfeeding has been well described so far (Bergstrom et al., 2014; Wampach et al., 2017; Bittinger et al., 2020), but just a study characterized the transition from exclusive breastfeeding to the introduction of complementary feeding in modulating the microbiota composition (Differding et al., 2020). Moreover, the mother’s fucosyltransferase 2 (FUT2) genotype, the Lewis histo-blood group antigens and the qualitative content of human milk oligosaccharides (HMOs) are also key factors in the establishment of the gut microbiota (Bai et al., 2018). HMOs are host-indigestible bioactive molecules that contribute to shape the gut microbiome by acting as prebiotics to favor beneficial microbes in the infants’ gut (Milani et al., 2017; Boudry et al., 2021). Some studies have applied an NMR-based metabolomics approach to investigate breast milk composition and its changes in relation to the lactation time, as well as to assess the mother’s *Secretor* genotype (Praticò et al., 2014; Cesare Marincola et al., 2015; Dessì et al., 2018). Stewart and colleagues divided the microbiota development into three distinct phases of progression: a developmental phase (months 3–14), a transitional phase (months 15–30) and a stable phase (months 31–46) (Stewart et al., 2018). During the first two phases, the composition of microbiota inhabitants changes significantly. Over the third phase, the microbial components seem to gain a more stable structure (Stewart et al., 2018). The change in gut microbiota shaping reflects a shift in metabolism of the infants’ gut (Di Mauro et al., 2013). The high degree of inter-individual variability still makes unclear how a variation in its composition may modify the functionality of the infant’s microbiome immediately at birth and for the next years of age, (Fouhy et al., 2012; Madan et al., 2012). Indeed, the main microbiota metabolic activities result from the cooperative, synergic, syntrophic, agonist and antagonist interactions of diverse microbial species, with a special focus on the links between the diet and the gut microbiota and between the microbiota metabolic activity and host metabolism (Holmes et al., 2012; Postler and Ghosh, 2017). Gut microbiota metabolic functions are mainly performed via diverse array of metabolites originating 1) from the transformation of residual dietary compounds that escape digestion in the upper gastrointestinal tract, 2) from compounds released from flaked enterocytes, 3) from the transformation of host-produced metabolic intermediates, 4) from *de novo* synthesis by gut microbes (Postler and Ghosh, 2017). The investigation of the metabolite levels in infants stool samples during lactation allows to disentangle the complexity of the gut microbiota evolution during the first stage of life, in relation to neonate diet. The aims of the longitudinal study were to follow the dynamics of an infant fecal microbiota and metabolome in relation to the breast milk composition during the lactation period, then to evaluate the changes induced by the introduction of complementary food during the weaning. We then focused on the gut microbiota developmental phase, where the transition from breastfeeding to weaning turned out to be a deterministic factor of the microbiota’s structure (Stewart et al., 2018). The relationship among diet, fecal microbiota composition and metabolism can be more easily identified by means of NMR-based metabolomics coupled with and 16S rRNA-based metagenomics on a single mother-infant dyad. Indeed, the choice to study multi-samples from a single mother-infant dyad avoids the superimposition of inter-variability onto individual intra-variability, allowing to disentangle the intricate dynamic involving diet, microbiota composition and metabolites. To that end, a multi-block (data integration) approach was applied to extract the maximum information from both the metabolomics and taxonomical metagenomics experimental data-blocks.

## 2 Materials and Methods

### 2.1 Sample Collection

A mother-infant dyad was recruited for a non-interventional study and followed-up from day 103 to day 268 after the neonate’s birth, in order to assess the fecal metabolite levels during a pure breast milk until a mixed milk/semisolid food diet. In this longitudinal study, breast milk and neonatal stool samples were collected in parallel from day 103 to day 108, from day 145 to day 147 and from day 165 to day 175 after the birth. Stool samples were still collected from day 219 to day 228 and from day 262 to day 268 after birth. The dataset for stools is composed by a total of 32 samples gathered in five sets of consecutive days and subjected to metabolomics (*n* = 32/32) and taxonomical metagenomics (*n* = 25/32) investigations (Supplementary Table S3). Three diet-related groups were formed: the “BM period” corresponding to breastfeeding (sets 103–108 and 145–147 days), the “FBM period” corresponding to the introduction of a minimal dose of complementary food (set 165–175 days) and the “W period,” corresponding to weaning (sets 219–228 and 262–268 days).

The peculiar experimental design of the study allowed to verify reproducibility of the observations within sets of consecutive days and to separate the possible variations due to the microbiota adaptation from those induced by the diet.

The 30-year-old mother and her infant were healthy. The infant was born at term by vaginal delivery. The mother-infant dyad did not take any antibiotic therapy for the 24 months preceding the collection of the first sample. The medical history of the two subjects was registered and no major disorders referred. The qualitative details of the infant diet over these time intervals are reported in Supplementary Table S3. In summary, for the first 147 days after birth baby was fed only by breast milk. Around the day 165, a fruit snack based on smashed raw pear or apple was daily added to the breast milk diet. Then, a meal based on vegetable stock (fresh chard, zucchini, carrots and string beans) enriched with tapioca or rice cream was added to the day 173. Subsequently, a homogenized meat meal dressed with Parmigiano Reggiano aged 30 months was introduced to the diet. Starting from the seventh month of baby life, the breast milk intake was reduced to single breastfeeding while two semi-solid meals (lamb and beef), based on meat, fish (sole and sea bass), vegetables, eggs, cheese (basically ricotta cheese) and fruits were consumed now on.

As a non-interventional study, the study was approved by the Ethics Committee of the “Policlinico Hospital” of Bari (study number 5908). The mother provided written informed consent prior study beginning.

### 2.2 Sample Preparation

#### 2.2.1 Nuclear Magnetic Resonance-Based Metabolomics

The breast milk extraction procedure for the metabolomic analysis was performed as previously described (Praticò et al., 2014). Briefly, 1 ml of breast milk was vortexed in 4 ml of a methanol–chloroform mixture (1:1 v/v) in polypropylene tubes and kept overnight at 4°C. Polar and organic phases were separated by centrifugation at 10,000 × g at 4°C for 20 min. The polar and the organic phases were separately collected, dried under N_2_ stream and preserved at −80°C until the subsequent analysis. The dried polar samples were re-dissolved in 600 μl of D_2_O containing 2 mM (final concentration) of 3-(trimethylsilyl)propionic-2,2,3,3-d_4_ acid sodium salt (TSP) (Sigma-Aldrich, St. Louis, MO, United States) as an internal reference. Five mm NMR glass tubes were used for the NMR analysis.

Fecal waters were obtained by adding 1 ml of PBS-D_2_O with 0.3% (final concentration) of sodium azide to 500 mg of the infant’s frozen feces. The samples were thawed for 30 min at room temperature and then vortexed to achieve a homogenous solution. The fecal waters were separated from their solid phase by a first centrifugation at 10,000 × g at 4°C for 25 min, hence filtered on a 40 μm pores filter. Two-hundred μl of PBS-D_2_O with 0.3% of sodium azide were added to the samples and centrifuged again at 10,000 × g at 4°C for 25 min. After withdrawing 600 μl of supernatant, 60 μl of PBS-D_2_O containing TSP (2 mM final concentration) were added. The samples were preserved at −80°C until the subsequent analysis. NMR spectra were acquired using a Bruker Avance III 400 spectrometer (Bruker BioSpin GmbH, Karlsruhe, Germany) equipped with a 9.4T magnet operating at ^1^H frequency of 400.13 MHz and at 298°K. Signals assignment was achieved by bidimensional experiments (COSY, TOCSY, HSQC, HMBC and DOSY) on selected samples and confirmed by comparison with literature (Jacobs et al., 2008; Praticò et al., 2014; Del Chierico et al., 2015; Dessì et al., 2018), web database (Wishart et al., 2012) and in-house database. One-dimensional (1D) NMR spectra were processed and quantified by using ACD/Lab 1D NMR Manager ver. 12.0 software (Advanced Chemistry Development, Inc., Toronto, ON, Canada), whereas bi-dimensional (2D) NMR spectra were processed by using Bruker TopSpin ver.3.1 (Bruker BioSpin GmbH) and MestreC ver.4.7.0.0 (Mestrelab Research SL, Santiago de Compostela, Spain). Phase and baseline of acquired NMR spectra were manually corrected. Quantification of metabolites was carried out by comparing the integrals of the resonances with the TSP signal integral and normalized for the number of protons.

#### 2.2.2 16S rRNA Gene Profiling

DNA was extracted from stool samples using QIAamp Fast DNA Stool mini kit (Qiagen, Germany), following the manufacturer’s instructions. The variable region V3-V4 of the 16S rRNA gene (∼460 bp) was amplified using the primer pairs reported in the MiSeq rRNA Amplicon Sequencing protocol (Illumina, San Diego, CA, United States). The amplicons were purified from primes and primer dimers by AMPure XP beads (Beckman Coulter Inc., Beverly, MA, United States). A second step of amplification was performed to attach a unique combination of Bar-coded Illumina Nextera forward and reverse adaptor-primers to amplicons of each sample. After a second purification step by AMPure XP beads, each DNA library (630 bp) was quantified using Quant-iT™ PicoGreen® dsDNA Assay Kit (Thermo Fisher Scientific, Waltham, MA, United States) and diluted to a final concentration of 4 nM. All libraries were pooled together and sequenced on a MiSeqTM instrument, by MiSeq Reagent Kit v2 (500 cycle) (Illumina), according to Illumina’s instructions. Operational Taxonomic Units (OTUs) tables were obtained by binding sequences into clusters with a 97% of pairwise identity and representative sequences were aligned using PyNAST v.0.1. software against Greengenes 13_08 database with a 97% of sequence similarity, Qiime 1.9.0 software (DeSantis et al., 2006; Caporaso et al., 2010). All raw sequences have been archived in NCBI database: PRJNA719939 (https://www.ncbi.nlm.nih.gov/bioproject).

### 2.3 Data Analysis and Statistics

To evaluate the differences in the milk and fecal metabolic profiles during the period under study, multivariate and univariate analyses were applied to the metabolite and OTUs matrices. Principal Component Analysis (PCA) was used to highlight possible clusters, to identify outliers and significant metabolites. All data were autoscaled before further data processing.

#### 2.3.1 Classification Using Partial Least Squares Discriminant Analysis

For the classification stage, different models were built using the Partial Least Square-Discriminant Analysis (PLS-DA) algorithm (Geladi and Kowalski, 1986; Ståhle and Wold, 1987) on stools grouped on the basis of breast milk only or weaning diets. Since PLS-DA is a predictive model, a validation phase is needed in order to evaluate the reliability of its prediction and, as a consequence of the candidate biomarkers suggested. To this purpose, in the present study an approach based on repeated double cross-validation (rDCV) coupled to permutation tests was followed (Westerhuis et al., 2008; Biancolillo et al., 2019). The term repeated indicates that, in order to avoid that the outcomes are based on a single data splitting scheme, the whole procedure is repeated a sufficiently large number of times (here 50): this approach allows also to estimate confidence intervals for the model predictions and stability/consistence of candidate biomarkers, which are in fact evaluated on the basis of the Rank Product (RP). To calculate rank product, at each DCV iteration, the predictors are ranked according to their contribution to the PLS-DA model (estimated as absolute value of the associated model coefficient), the one contributing the most being given rank 1 and so on. At the end of the rDCV procedure, for each variable the geometric average of its ranks across all the iterations is defined as the rank product index. Low values of RP indicate variables highly contributing to the model and, accordingly, candidate biomarkers. Finally, to rule out any possibility that good classification results could be obtained by chance, permutation tests are used to non-parametrically estimate the distributions of the classification figures of merit under the null hypothesis for significance testing. In the present study, each permutation test was carried out based on 1,000 randomizations. Three figures of merit were used to summarize the quality and the predictive ability of the classification model, namely the number of misclassifications (NMC), the area under the receiver operating characteristic curve (AUROC) and the discriminant Q^2^ (DQ^2^). The number of misclassifications is the number of samples which are wrongly classified by the model and it is therefore inversely related to the predictive ability. For a binary classifier, the ROC curve is a way of displaying how the sensitivity and the specificity of the model vary as a function of the discriminant threshold: the closer the area under the ROC curve is to 1, the better and more accurate the classification model. Lastly, the discriminant Q^2^ is a figure of merit which was introduced for regression-based classification models, such as PLS-DA: it is defined analogously to classical *R* ^2^ but residuals are differentially weighted depending on whether they lead to a correctly or incorrectly classified sample. For further interpretation and considerations, the metabolites highlighted as relevant on the basis of their RP value were considered. Two tailed Student’s t-test was applied to assess the differences on the metabolite levels between two milk groups: at the beginning and at the end of the breastfeeding. *p* value lower than 0.05 was considered significant.

#### 2.3.2 Omics Data Integration

Since data were obtained by NMR-based metabolomics and taxonomical metagenomics, to extract the maximum information from the experimental outcomes, a multi-block (data integration) approach was followed (Qannari et al., 2000). Indeed, in multi-block data analysis, the matrices collecting the experimental data from the different techniques (data blocks), rather than being processed individually, are jointly elaborated, so to highlight more clearly correlations between metabolites and OTUs, information which is common between the two platform and, also, information which is carried uniquely by each of them. In particular, in the present study both an exploratory (unsupervised) and a predictive (supervised) analysis were carried out, by means of Common component and specific weight analysis (CCSWA, most commonly referred as ComDim; (Qannari et al., 2000); and multi-block partial least squares discriminant analysis (MB-PLSDA), respectively.

#### 2.3.3 Common Components and Specific Weight Analysis (CCSWA, ComDim)

ComDim is an exploratory multi-block method which can be considered as one of the possible generalizations of principal component analysis for the case when multiple data matrices should be simultaneously processed (Qannari et al., 2000). In particular, ComDim aims at extracting components which explain as much joint (common) variability between the data blocks as possible. This results in generating a set of scores **T**, which reflect the overall similarities/dissimilarities among the samples based on the simultaneous analysis of all the data matrices. These scores can, as in normal PC, be graphically displayed in two- or three-dimensional plots to provide a visual and immediate representation of the relationships among the samples. The relative contributions of the blocks to the individual components, which allow to identify to what extent the various component summarize information which is common among the platforms or unique to some specific blocks, are called saliences and indicated as λ_ij_, i being the component and *j* being the block. On the other hand, the common set of scores **T** can be projected onto the individual data matrices to obtain an associated set of loadings **P**_j_ for each of the data blocks under investigation, which allow to interpret the relationship between the samples (e.g., as shown in a scores plot) in terms of the measured variables.

#### 2.3.4 Multi-Block Partial Least Squares Discriminant Analysis

Multi-block PLS-DAMB-PLSDA (Qannari et al., 2000) consists in building a PLS-DA model after low-level data integration, i.e. on a data set obtained by concatenating the experimental matrices corresponding to the different blocks of data (usually, after scaling each block by its Frobenius’ norm, so to “equalize” their relative contribution). Validation of the MB-PLSDA model by means of repeated double cross-validation and identification of candidate biomarkers based on the values of the rank product index were carried out as described for single block PLS-DA in *Classification Using Partial Least Squares Discriminant Analysis*.

## 3 Results

The mother’s body mass index shifted from 24 at day 103 to 22 at day 268. Both weight and length of the baby ranged within the 75th and 50th percentile during the follow-up period.

### 3.1 Breast Milk Metabolic Profiles

The NMR metabolic profiling of the breast milk samples revealed a total of 39 metabolites identified and quantified. The ^1^H chemical shifts, multiplicity, ^13^C chemical shifts and assignments are reported as supplementary material (Supplementary Table S1). To detect the differences in milk composition between the beginning and the end of suction, we compared paired samples collected at both of these points. The result showed a significantly higher content of lactate (*p* = 0.007) at the end of suction (Supplementary Figure S1). Then, the following analyses were performed on breast milk samples collected at the end of suction. To assess the evolution of milk composition as a function of lactation time, expressed as days after the birth, PLS analysis was carried out. The PLS model showed six significant latent variables, with *R* ^2^Y = 0.99 and Q^2^Y = 0.91. The regression coefficients analysis showed a significant increase (*p* < 0.05) of 3′-fucosyllactose (3′FL) levels and a significant decrease of N-acetyl moieties of oligosaccharides, dimethylamine (DMA), lacto-N-fucopentaose III (LNFP III), 2′-fucosyllactose (2′FL) and lacto-N-fucopentaose I (LNFP I) levels (Supplementary Figure S2).

### 3.2 Infant Gut Microbiota Metabolic Profiling

**FIGURE 1:**
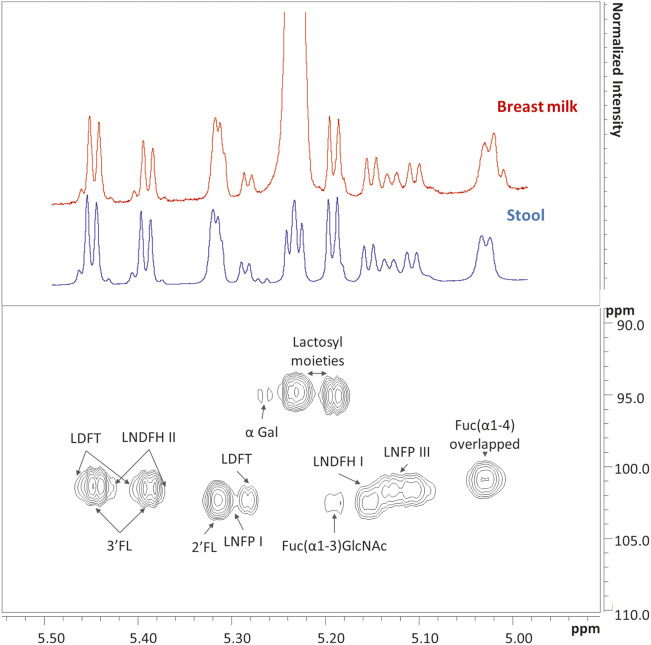
In the upper panel are showed two representatives ^1^H NMR spectra of breast milk (red) and infant’s stool (blue) collected at day 104 after birth, as focused on the human milk oligosaccharides (HMOs) anomeric region from 5 to 5.50 ppm. The corresponding Heteronuclear Single Quantum Correlation (HSQC) experiment of the stool sample is showed in the lower panel. In the upper panel, the lactose resonance of breast milk is the highest in intensity. To better show that most of the metabolites are shared in the two biological matrices at this specific time point, a cut-off has been defined and both spectra have been scaled in intensity. List of abbreviations: α-Gal, α-galactose; Fuc, fucose; GlcNAc, N-acetylglucosamine; 2′FL, 2′fucosyllactose; 3′FL, 3′fucosyllactose; LDFT, lactodifucotetraose; LNDFH I, II, lacto-N-difucohesaose I, II; LNFP I, III, lacto-N-fucopentaose I, III.

**FIGURE 2:**
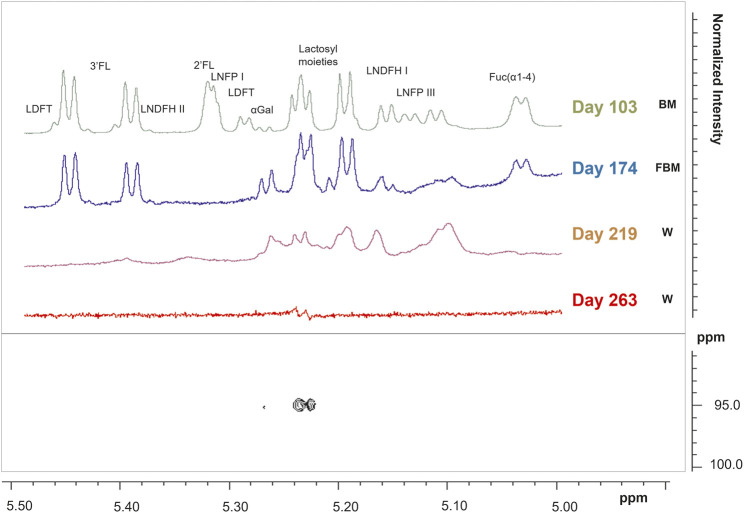
^1^H NMR spectra of infant’s stool samples as focused on the human milk oligosaccharides (HMOs) anomeric region from 5 to 5.50 ppm. Spectra of samples collected at days 103, 174, 219, and 263 after birth were reported in the upper panel. A representative Heteronuclear Single Quantum Correlation (HSQC) experiment of a fecal sample collected at the last sampling point, corresponding to day 263, showed how the lactosyl resonance is the only left among the other HMOs resonances (lower panel).

**FIGURE 3:**
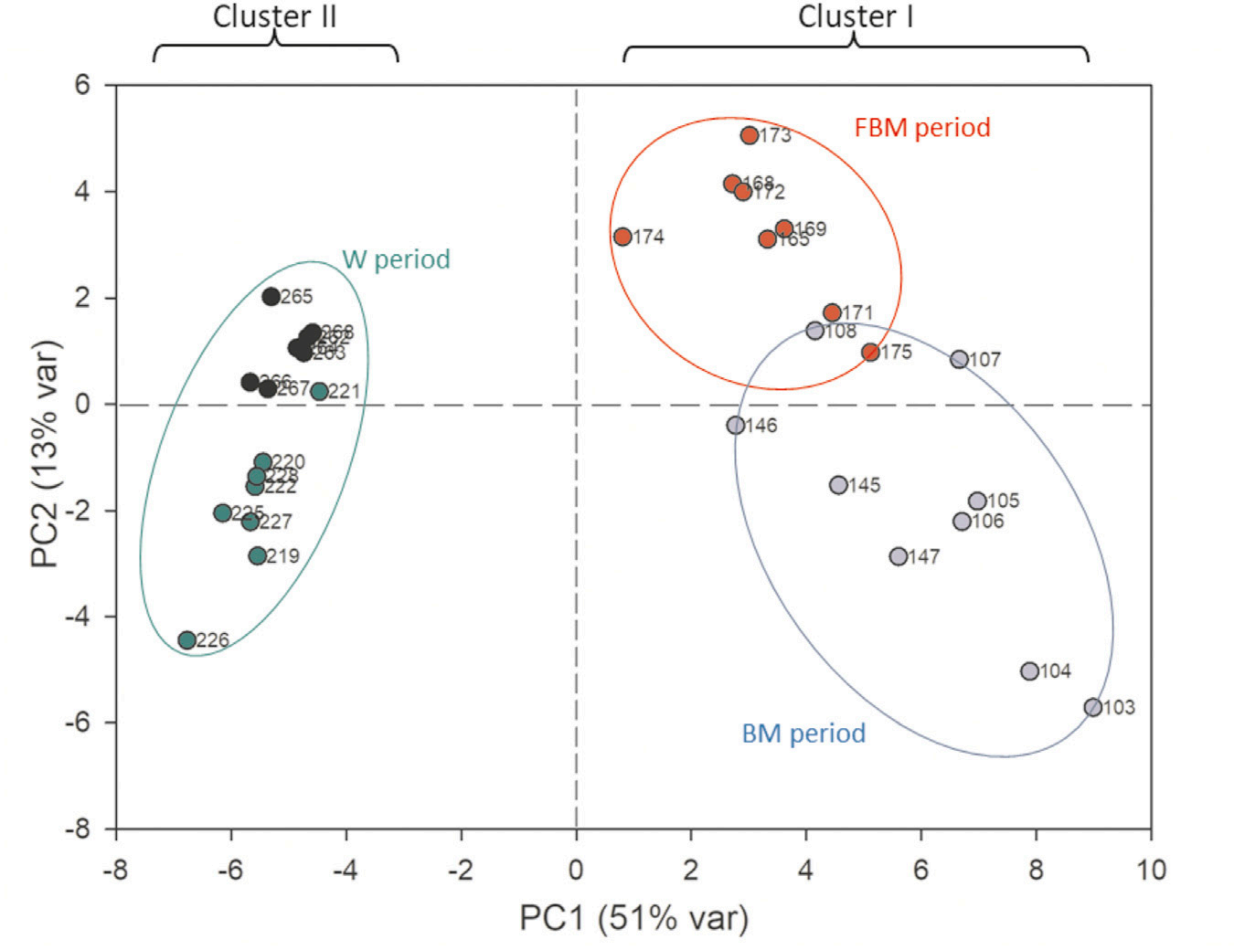
PCA scores plot performed on the metabolomic dataset of stool samples. The first principal component (PC1) accounts for the 51% of the overall variance, while the second (PC2) accounts for the 13%. Blue dots: 103–147 days; red dots: 165–175 days; green dots: 219–228 days; black dots: 262–268 days.

In stool samples, a total of 61 metabolites were identified, 49 of which were quantified (Supplementary Table S1). Figure 1 shows the comparison between two representative breast milk and stool NMR spectra, corresponding to a milk-based diet time point (day 104 after birth). The pattern of human milk oligosaccharide resonances appeared mainly preserved in the stool spectrum. However, the CH-1 HMOs lactosyl moieties (5.23 ppm) signal was barely visible in the stool spectrum due to the disappearance of the signal of lactose. The CH-1 α-galactose (α-Gal) resonance was only present in the stool spectrum. The signal at 5.02 ppm (possibly related to a fucosyl-moiety) was almost undetectable in stool samples. Four representative NMR spectra were reported in Figure 2 to show the evolution of the HMOs trend in stools due to the infant’s gut microbiota activity throughout the time. From day 103 to day 174, the signals assigned to 2′fucosyllactose (2′FL), lacto-N-fucopentaose I (LNFP I) and lactodifucotetraose (LDFT) disappeared and only lower levels of lacto-N-difucohexaose I, II (LNDFH I, II) and lacto-N-fucopentaose III (LNFP III) were observed. The pattern of HMOs in the stool spectrum at day 219 changed again showing the disappearance of the 3′fucosyllactose (3′FL), lacto-N-difucohesaose I, II (LNDFH I, II), LNFP III and fucosyl (α1-4) resonances. As shown in the HSQC spectrum of day 263 sample, the lactosyl moiety cross-peak was the only still present, although barely visible, whilst everything else disappeared over time. In order to explore stools data, a PCA was performed on the metabolite concentrations of 32 samples. The PCA model showed two principal components (PC1 and PC2) accounting for 51 and 13% of the overall variance, respectively (Figure 3). We can easily observe two main clusters along PC1: samples from days 103–175 and samples from days 219–268. A further clustering could be observed along PC2 axis (Figure 3), still related to the infant’s diet.

**FIGURE 4:**
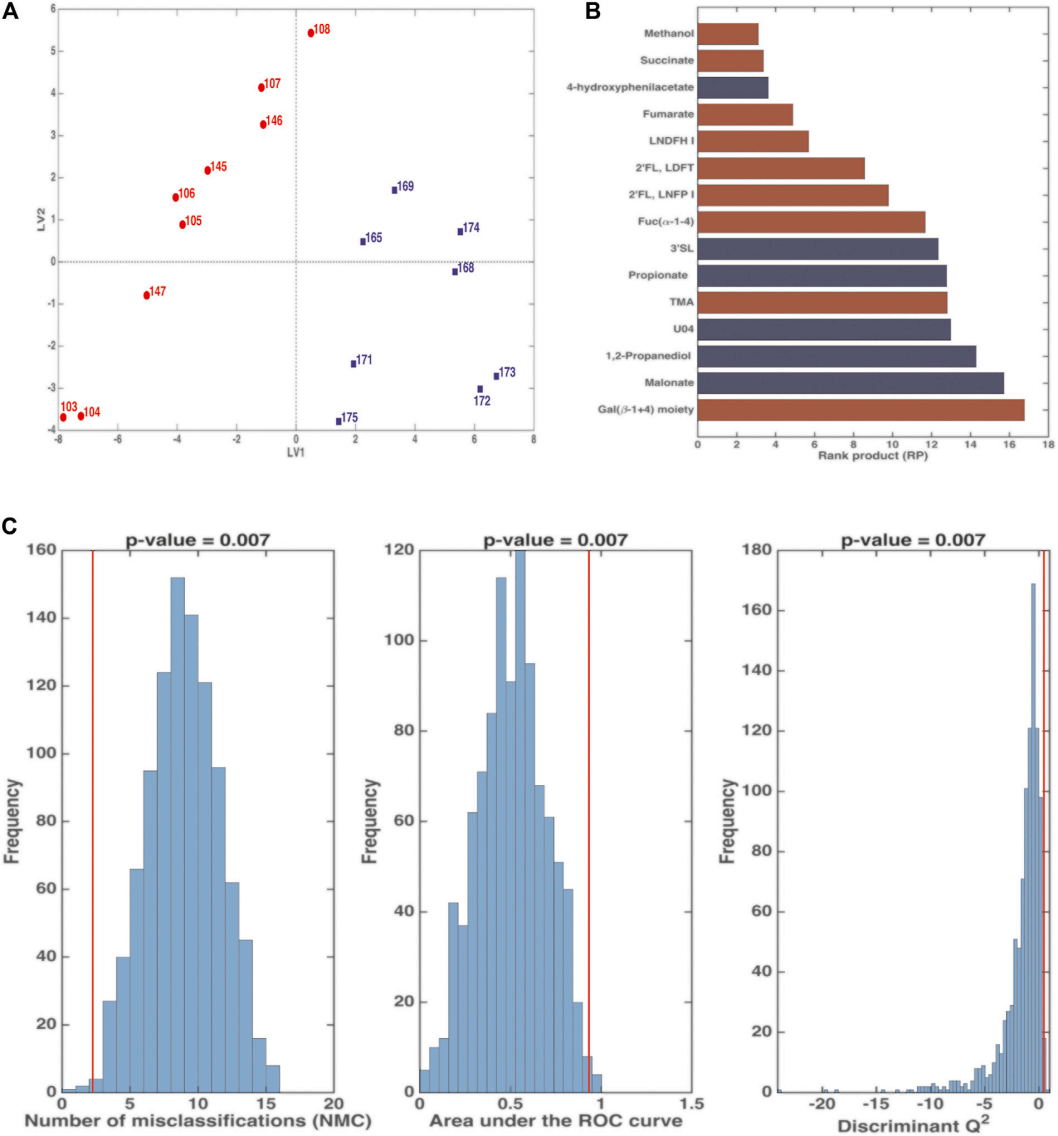
PLS-DA analysis in Double Cross Validation (DCV) of stool samples on BM (red) and FBM (blue) periods. Panel **(A)** reports latent variables (LV) scores plot. Panel **(B)** reports the variables that significantly contributes to the PLS-DA model by means of the rank product (RP). The criterion RP <average RP was used to identify the potential markers, that are the ones displayed as bar plot in the panel. Red variables were higher in the BM period; blue variables were higher in the FBM period. Panel **(C)** reports the validation of the PLS-DA model. The figures of merit namely Number of Misclassifications (NMC), Area Under the Receiver Operating Characteristic curve (AUROC) and Discriminant Q^2^ (DQ^2^), calculated on the outer cross-validation loop (red vertical bars) were compared to their respective distribution under the null hypothesis (estimated by means of permutation test), indicating that the discrimination observed is statistically significant.

The PC1 loadings allowed to characterize these two clusters in relation to the diet, indeed the first one was characterized by higher values of HMOs and glycolytic intermediates, such as lactate and succinate, whilst the second one was characterized by higher values of short chain fatty acids (SCFA) (i.e., acetate, butyrate and propionate), branched (i.e., valine, isoleucine) and aromatic (i.e., phenylalanine, tyrosine) amino acids, intermediates of aromatic amino acids, ethanol, methylamine compounds (i.e., trimethylamine, methylamine) and nicotinamide (data not shown). To explore the data distributions more in the details, two PLS-DA models were separately calculated on the first cluster (Figure 4) as well as on the second cluster (Supplementary Figure S5).

Figure 4 shows that the first cluster distinguishes between the BM period (days 103–108 and 145–147) and the FBM period (days 165–175). The significant RP values of are reported in Figure 4B. FBM stool samples showed high levels 4-hydroxyphenylacetate (4-HPA), 3′SL, propionate, U04, 1,2-propanediol (1,2-PD), malonate and low levels of fucosyl-oligosaccharides as compared to BM samples. Intriguingly, methanol and succinate levels in FBM samples were lower than BM ones. The significance of the PLS-DA model in DCV is witnessed by *p* = 0.007 value for NMC, AUROC and DQ^2^ (Figure 4C). The total correct classification rate (ccr) was 87 ± 4% (ccr = 91 ± 7% and 82 ± 8% for BM and FBM, respectively).

Supplementary Figure S3 shows the histograms of the time-dependent changes in stools metabolite levels during the five examined sets of consecutive days, highlighting interesting trends of variation. Metabolite levels showed either a increasing (i.e., SCFA, amino acids, ethanol, biliary salt 2) or decreasing (i.e., oligosaccharides, succinate) trend, related to the different period under study. Intriguingly, biliary salt 2 displays a tendency which differs from that of the biliary salt 1 (Supplementary Figure S3). Their resonances represent the protons linked to the C18 of unconjugated and conjugated biliary salts. Comparing the levels of biliary salt 1 (0.67 ppm) and biliary salt 2 (0.73 ppm), a similar trend is shown from day 103 to day 175 for both species but, starting from day 219 to day 268, an increase in levels of some particular species of biliary salt 2 appeared. This might be the deoxycholic salt and its glycine and taurine conjugates, connected to the weaning period. Only few metabolites exhibited a peculiar trend in specific periods. Formate and 1,2-PD are some of those, increasing their levels just in the timeframe between day 165 and day 175, concurrently with the addition of a fruit snack to an exclusive milk-based diet (FBM period).

**FIGURE 5:**
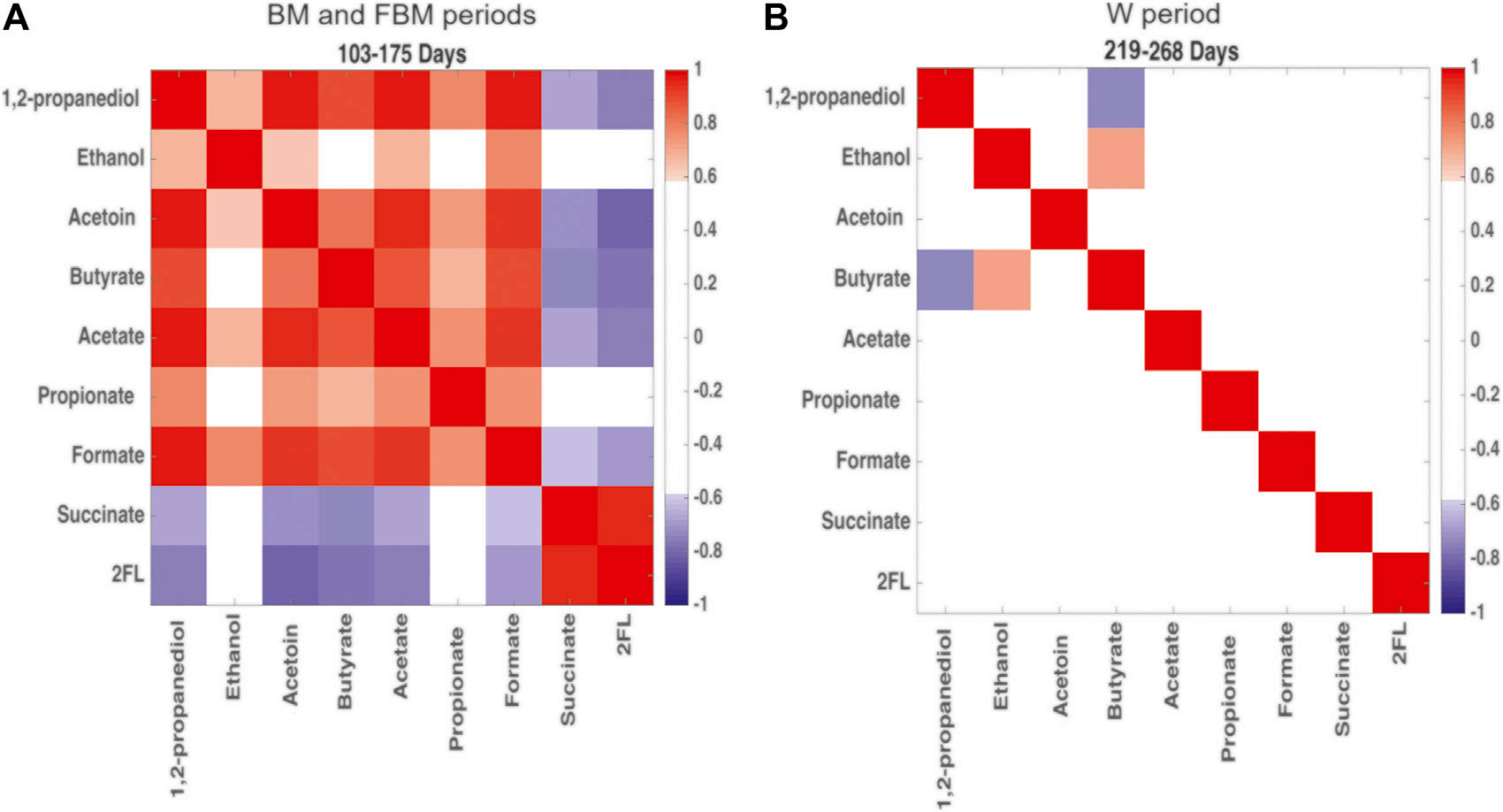
Heatmap built from Pearson’s correlation coefficients for the microbial metabolic products of carbohydrate degradation. Comparison between **(A)** days 103–175, predominantly breastfeeding, and **(B)** days 219–268, predominantly solid food. Red: positive correlations; blue: negative correlations. Correlation coefficient >0.6 and < −0.6 are significant with *p* < 0.05.

The Pearson’s correlation matrix restricted to 1,2-PD, ethanol, acetoin, butyrate, acetate, propionate, formate, succinate, and 2′FL (as representative of the oligosaccharide class) was built as a heatmap in order to detect changes in the metabolite correlation network occurring during the two main diet-dependent periods: 103–175 days and 219–268 days (Figure 5). Positive correlations among 1,2-PD, ethanol, acetoin, butyrate, acetate, propionate and formate were showed at 103–175 days (Figure 5A); succinate was positively correlated with 2′FL but they were both negatively correlated with the other metabolic products. At 220–268 days mostly all of the correlations between fermentation products disappeared. Only a positive correlation between butyrate and ethanol and a negative correlation between butyrate and 1,2-PD were still observed (Figure 5B).

### 3.3 Infant Gut Microbiota Bacterial Composition

**FIGURE 6:**
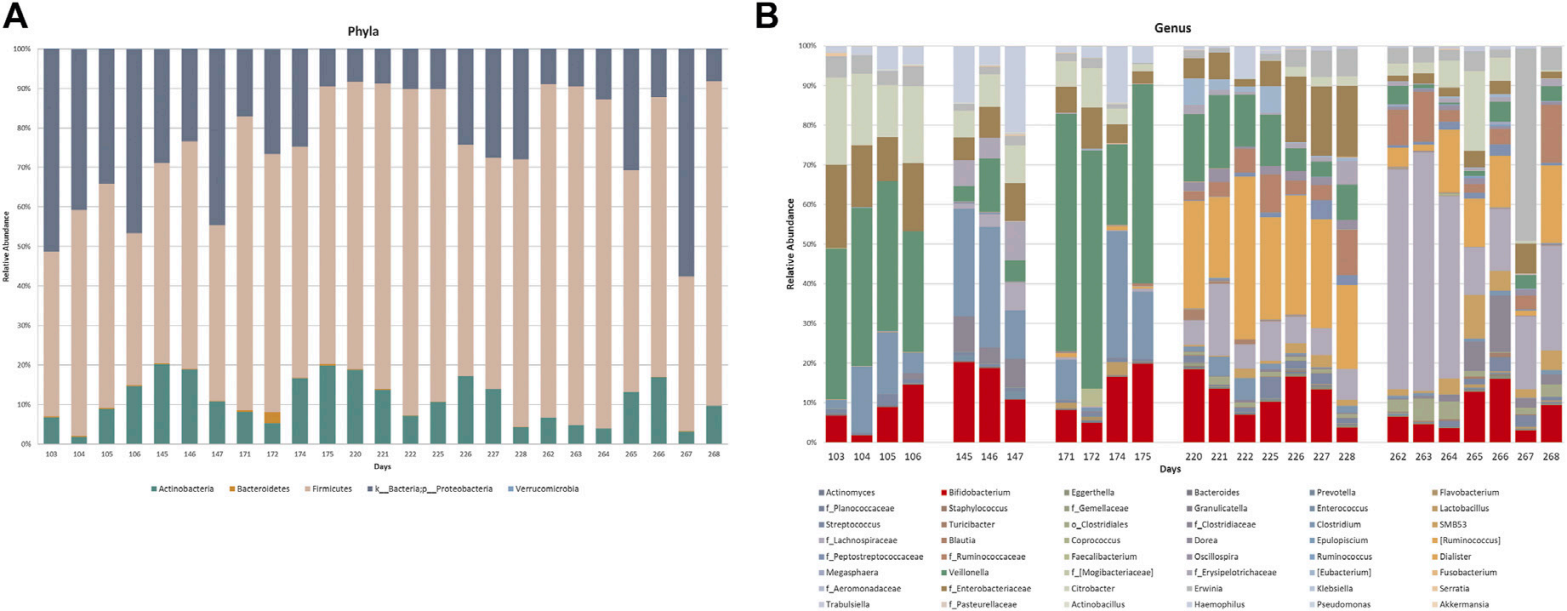
Relative abundance of OTUs at phylum **(A)** and genus **(B)** levels of the gut microbiota profiling. Where the genus level was not detectable, order (o_) or family (f_) was reported.

Targeted taxonomical metagenomics was performed on 25 stool samples (out of a total of 32) collected in five sets of consecutive days: 103–106, 145–147, 171–175, 220–228, and 262–268 days after birth. The relative abundances of OTUs at phylum level, expressed as percentage, are showed in Figure 6. The infant enterotype was characterized by high levels of Firmicutes, Proteobacteria and Actinobacteria (Figure 6A). Bacteroidetes were lower than 1% abundance in most of these days. Results did not show significant changes from day 103 to day 268.

Figure 6B shows the relative abundance of OTUs at genus-level. OTUs exceeding 1% abundance were included in the study, hence only 48 out of the 256 OTUs analyzed were considered. The selection was made by using an identity threshold >80%. The analysis of the qualitative data highlighted a high, albeit fluctuating, abundance of Bifidobacteria from day 103 to day 268. On the contrary, important variations in the abundance of other OTUs were observed. Changes between days 103–106 and 145–147 could not be ascribable to a different feeding. For instance, *Veillonella* (green section) and f_Enterobacteriaceae (brown section) abundance variations occurred during the breastfeeding period (BM), when no diet change occurred. On the other hand, a characteristic variation in the microbiota composition occurred along with the transition from a diet mainly based on breast milk to weaning: from day 220 to day 268 high abundance of *Ruminococcus* belonging to the family of Lachnospiraceae (light blue section), g_Lachnospiraceae (violet section), and unclassified Ruminococcaceae (orange section) were observed. However, these patterns were unstable even whether the diet did not change. The trend of some OTUs was reported as histograms for the five examined sets of consecutive days in Supplementary Figure S4.

**FIGURE 7:**
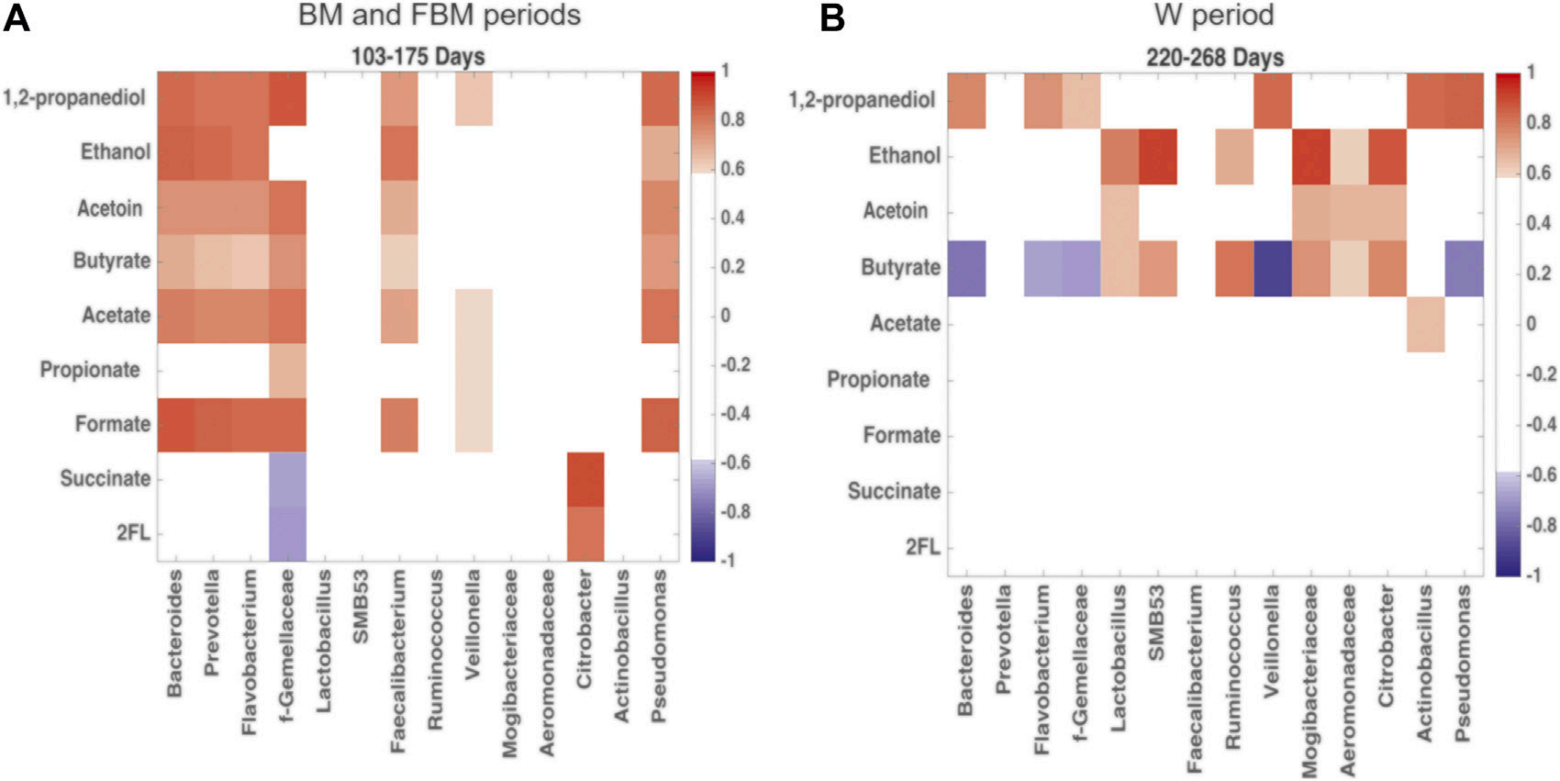
Heatmap built from Pearson’s correlation coefficients for the microbial metabolic products of carbohydrate degradation and OTUs. Comparison between **(A)** days 103–175, predominantly breastfeeding, and **(B)** days 220–268, predominantly solid food. Red: positive correlations; blue: negative correlations. Correlation coefficient >0.6 and < −0.6 are significant with *p* < 0.05. Only OTUs with significant correlation coefficient for carbohydrate degradation products were reported in figure.

The changes between microbial products and OTUs during the transition from exclusive breastfeeding to weaning can be disentangled through the Pearson’s correlation matrix analysis, reported in the heatmap in Figure 7. In Figure 7A, 1,2-PD, ethanol, acetoin, butyrate, acetate and formate levels were positively correlated with Bacteroidetes and Firmicutes genera (known butyrate producers), including *Bacteroides*, *Prevotella*, *Flavobacterium*, unclassified Gemellaceae, *Faecalibacterium* and *Veillonella*. A positive correlation with *Pseudomonas*, belonging to *Proteobacteria*, was also observed. The oligosaccharides, represented by the metabolite 2′FL, were negatively correlated with these OTUs, even though statistical significance was not achieved. In the weaning period (Figure 7B) the previous correlation network strongly changed, indicating just a positive correlation between those OTUs and 1,2-PD. Correlation with butyrate levels became negative. On the other hand, butyrate-producing OTUs belonging to Firmicutes and Proteobacteria, such as *Lactobacillus*, *SMB53*, *Ruminococcus*, Mogibacteriaceae, unclassified Aeromonadaceae and *Citrobacter*, resulted positively correlated with butyrate, acetoin and ethanol.

### 3.4 Omic Data Integration: Metabolomics and Taxonomical Metagenomics of Stool Samples

**FIGURE 8:**
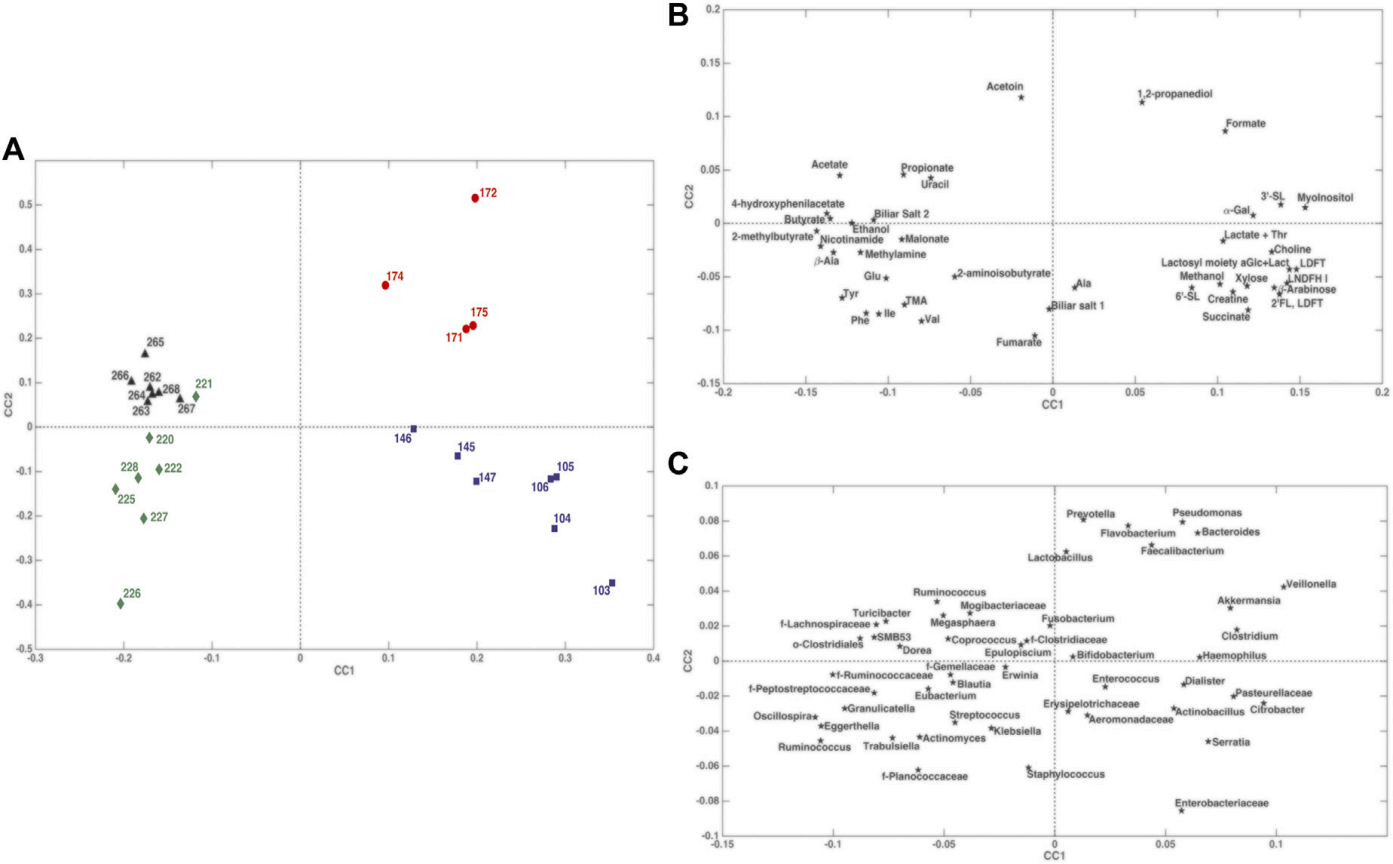
ComDim scores and loadings plots of the individual components built from metabolomics and taxonomical metagenomics data. Panel **(A)**: scores plot; panel **(B)**: loadings plot for the metabolomic data set; panel **(C)**: loadings plot for the metagenomic data set.

**FIGURE 9:**
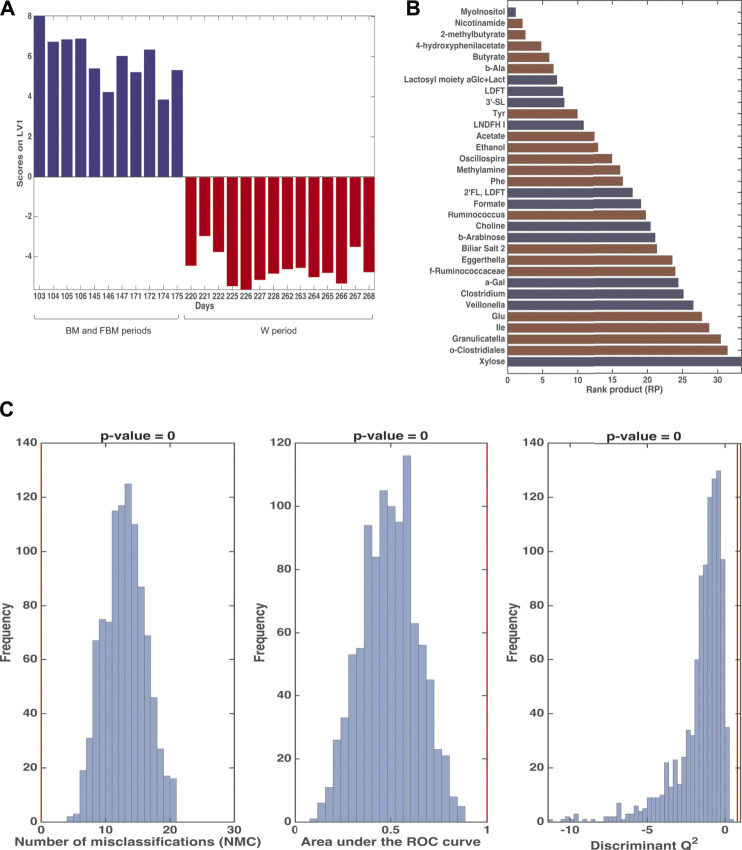
Results of Multiblock PLS-DA in repeated Double Cross Validation for the comparison between 103–175 days and 220–268. Panel **(A)**: plot of the mean scores of the outer loop samples along the only latent variable of the model (LV1). Blue: 103–175 days; red: 220–268 days. Panel **(B)**: significant variables for the PLS-DA model by means of the rank product (RP). The criterion RP <average RP was used to identify the potential markers, which are the ones displayed as bar plot in the panel. Blue: values higher in samples collected at 103–175 days; red: values higher in samples collected at 220–268 days. Panel **(C)**: validation of the PLS-DA model. The figures of merit namely Number of Misclassifications (NMC), Area Under the ROC curve and Discriminant Q^2^, calculated on the outer cross-validation loop (red vertical bars) are compared to their respective distribution under the null hypothesis (estimated by means of permutation test), indicating that the discrimination observed is statistically significant.

The ComDim model was applied in order to extract components that could account for as much common variability as possible between the metabolomics and taxonomical metagenomic data blocks. In particular, the model was calculated after individual auto-scaling of each data matrix and successive block-scaling (dividing each block by its Frobenius’ norm). Figure 8 shows the scores plot and the individual component loadings plots. For the first two components, the contribution to the individual components (salience) of the metabolome block was higher than that of the metagenome block (data not shown). This finding was confirmed by the CC score plot (Figure 8A) which showed a score distribution very similar to the one obtained with the PCA model performed on the single metabolomics matrix (Figure 3). Of particular interest is the overlapping between the loadings of *Bacteroides*, *Prevotella*, *Flavobacterium*, *Faecalibacterium*, *Pseudomonas* and *Veillonella* (Figure 8C) with 1,2-PD and formate (Figure 8B) in the quadrant corresponding to the samples collected from day 171 to day 175 days. The results of multiblock PLS-DA in DCV are graphically displayed in Figure 9. Also in this case, prior to model building, the individual data matrices were preprocessed by autoscaling followed by division by their respective Frobenius’norm, and successively concatenated row-wise. The mean scores of the outer loop (external validation) samples along LV1 and the metabolites with significant RP values are also given in Figures 9A,B, respectively. The model turned out to be highly significant at the permutation test (*p* < 10^−4^), as shown in Figure 9C, and predictive with 100% correct classification rate.

## 4 Discussion

In the present study, the longitudinal multi-omics investigation on a single mother-infant dyad was proposed to examine in depth the relationships among diet composition, gut microbiota metabolism and composition during the transition from breastfeeding to weaning. We focused on the time-course changes that occurred in breast milk composition and in the fecal metabolome of the newborn, analyzing its composition during the exclusive breastfeeding until the transition to semisolid food.

The experimental design of the study was meant to verify reproducibility of the observations within sets of consecutive days and to separate the variations due to the microbiota adaptation from those induced by feeding. In a previous study (Bäckhed et al., 2015), the stronger impact in shaping the gut microbiota composition was suggested to be due to the cessation of the breast-feeding, rather than the introduction of solid foods. In our study the breastfeeding was not completely stopped over time, even if largely reduced, guaranteeing a minimum intake of HMOs. Nevertheless, the gut microbiome changes occurred concurrently with the introduction of solid foods, as Bäckhed and colleagues also observed (Bäckhed et al., 2015), suggesting a more complex dynamic that involves the microbiota adaptation to a more diversified diet along with the host’s development.

Starting from the description of the microbiota dynamic progression of a single individual, the purpose of the work was not intent on providing a unique physiological reference model, but to make the main findings as generalizable and, also, to make the conceptualization of the multi-omic approach transferable to wider study-cases, either within the field of strategies for precision nutrition and/or treatments.

The maternal breast milk contained (α 1-2)-linked fucose HMOs (i.e., as 2′FL, LNFP I, LNDFH I and LDFT) thus confirming mother’s *Secretor* genotype (Praticò et al., 2014), which is common in many populations (Lewis et al., 2015). The intestinal degradation of these oligosaccharides is up to the ability of certain bacterial taxa to cleave the (α 1-2)-linked fucose, exposing the core of the oligosaccharides to the sequential enzyme attacks of other commensals (Lewis et al., 2015; Milani et al., 2017). High levels of fucosyl-oligosaccharides were observed in infant’s stools, identifying this trait as a not common occurrence (Del Chierico et al., 2015).

The consumption of fucosyl-(α 1-2)-oligosaccharides is commonly associated with an increased abundance of *Bifidobacterium* species (Bidart et al., 2014; Lewis et al., 2015). However, their efficient utilization is not exclusive for infant’s Bifidobacteria but it is strain-dependent and linked to the presence of ATP-binding cassette transporters (ABC) transporter (Matsuki et al., 2016).

Furthermore, many strains of *Bifidobacterium* are also reported to use Lacto-N-Tetraose (Sela et al., 2008). According to these findings, data collected from the BM period appeared to be in line with an increased abundance of Bifidobacteria non-consuming fucosyl-(α 1-2)-oligosaccharides. Moreover, CH-1 α-galactose resonance is only present in the stool spectrum, due to intestinal lactose hydrolysis. Despite the high *Bifidobacterium* spp. abundance, the galactose intracellular metabolism was not efficient enough because of the absence Tagatose pathway, in agreement with previous results (Wu et al., 2015). Concurrently with the intake of a fruit snack (FBM period), 2′FL, LNFP I, LNDFH I and LDFT levels dropped, thus suggesting the development of bacterial strains consuming fucosyl-oligosaccharides. Because of the breast milk intake per day was nearly constant, the decrease in fecal fucosylated HMOs cannot be explained just by the physiological reduction of the HMOs content. Although 16s rRNA profiling data could not prove variations at genus level, changes occurring in the FBM period for both 1,2-PD abundance and Bacteroidetes phylum suggested the development of fucosylated-HMOs consuming bacterial strains, possibly influencing the other commensals.

Through the exploration of the loadings plot of the two data blocks, the ComDim analysis allowed to point out the co-occurrence of *Bacteroides*, *Prevotella*, *Flavobacterium*, *Faecalibacterium*, *Veillonella* and *Pseudomonas* with an increasing in 1,2-PD and formate levels.

Co-fermentation experiments of oligosaccharides and fucosyl-oligosaccharides was found to promote 1,2-PD concentrations, suggesting a synergistic effect (Dedon et al., 2020). Its production has been related to the super-pathway of fucose degradation through the intra-cellular α-fucosidase activity and a series of phosphorylated intermediates (Boronat and Aguilar, 1981). *Bifidobacterium* strains i.e., *Bifidobacterium longum* ssp. *infantis* lack the genes encoding proteins to use fucose via phosphorylation (Sela et al., 2008), which are however possessed by other bacteria such as *Escherichia coli*. Moreover, some studies reported that the *Eubacterium hallii*, an early occurring commensal, can metabolize 1,2-PD to produce propionate, butyrate and formate, all metabolites which have positively impact on the trophic relationship within the infant gut microbiome (Schwab et al., 2017; Bunesova et al., 2018).

In line with these results, a recent glycomics and metagenomics analyses conducted on two infants showed a shift of fecal bacterial populations from HMOs-non-consuming to HMO-consuming bacteria during the first 13 weeks of life, associated with an increase in Bifidobacteriaceae and Bacteroidaceae (De Leoz et al., 2015). However, in the present study, the abundance of Bacteroidetes remained below the expected levels previously observed. The increase in 1,2-PD levels observed during the FBM period is likely to be ascribable to the fucose degradation pathway depending on the development of mutualist bacteria selected by fruit carbohydrates and polysaccharides. The correlation analyses on metabolite from BM and FBM samples showed a strong positive relation among 1,2-PD, SCFA, formate and a negative correlation between these metabolites and 2′FL, succinate. Ethanol was significantly correlated with acetate, acetoin and formate. Exploring the correlations among metabolites and OTUs in the same sample-sets, *Bacteroides*, *Prevotella*, *Flavobacterium*, *Faecalibacterium*, *Veillonella* and *Pseudomonas* appeared to be directly correlated with SCFA, acetoin, 1,2-PD, ethanol, and inversely correlated with 2′FL and succinate. These metabolites are involved in pyruvate catabolism carried out by the human gut microbes (Oliphant and Allen-Vercoe, 2019).

The correlation matrix confirmed a development of trophic interactions among bacterial species, particularly when fruits were added to the infant’s diet. With the introduction of complementary foods to the diet (three meals out of a total of five), these correlations reversed. At the beginning of weaning the infant ate a significant proportion of starch (tapioca and rice), plant metabolites (vegetable broth), animal proteins (lamb baby food) and fibers. Due to the physiological immaturity of the pancreatic exocrine function during this phase of growth, they could escape from the complete digestion reaching the colon microbiota, providing new substrates and promoting the dominance of novel bacterial communities. The transition from an exclusive breast milk-based diet to the introduction of semi-solid foods was well described by the PCA in Figure 3, where the separation along PC1 mainly reflected the effects of the diet composition, thus the transition from the HMOs metabolism to the metabolic pathways involving starch and protein degradation.

In the weaning period was observed a huge increasing in SCFA (acetate, propionate, butyrate) and ethanol, associated to the increase in amino acids and their intermediates, involved in protein fermentation (β-alanine and 4-hydroxyphenylalanine) and catabolism (branched SCFA).

High levels of biliary salt 2 appeared to be in agreement with the recent results obtained by Tanaka et al. on healthy Japanese infants during the first 3 years of life (Tanaka et al., 2020). They found out that the fecal unconjugated and conjugated deoxycholic acids were associated with an increment of Lachnospiraceae abundance and *Ruminococcus*, during the weaning (Tanaka et al., 2020).

The MB-PLSDA described the metabolites and the microbiota structure changes induced by the transition diet. The increase of SCFA, branched SCFA, amino acids and intermediates of protein fermentation was positively correlated to *Oscillospira*, *Ruminococcus* belonging to Lachnospiraceae, *Eggerthella*, unclassified o-Clostridiales and f_Ruminococcaceae abundances. Human observational studies found positive associations between *Lachnospira* abundance and consumption of fruits and vegetables (Herman et al., 2020). The fluctuation of the abundance of *Bifidobacterium* seemed to be independent of the feeding transition.

The end-products of the microbial anaerobic metabolism of fucosyl-oligosaccharides or monosaccharides derived from starch or pectins could act as an intermediate in a mixed mutualistic microbiota. Embden-Meyerhof-Parnas (EMP) glycolysis can be considered as the most important fermentation leading to the production of pyruvate and acetyl-CoA.

**FIGURE 10:**
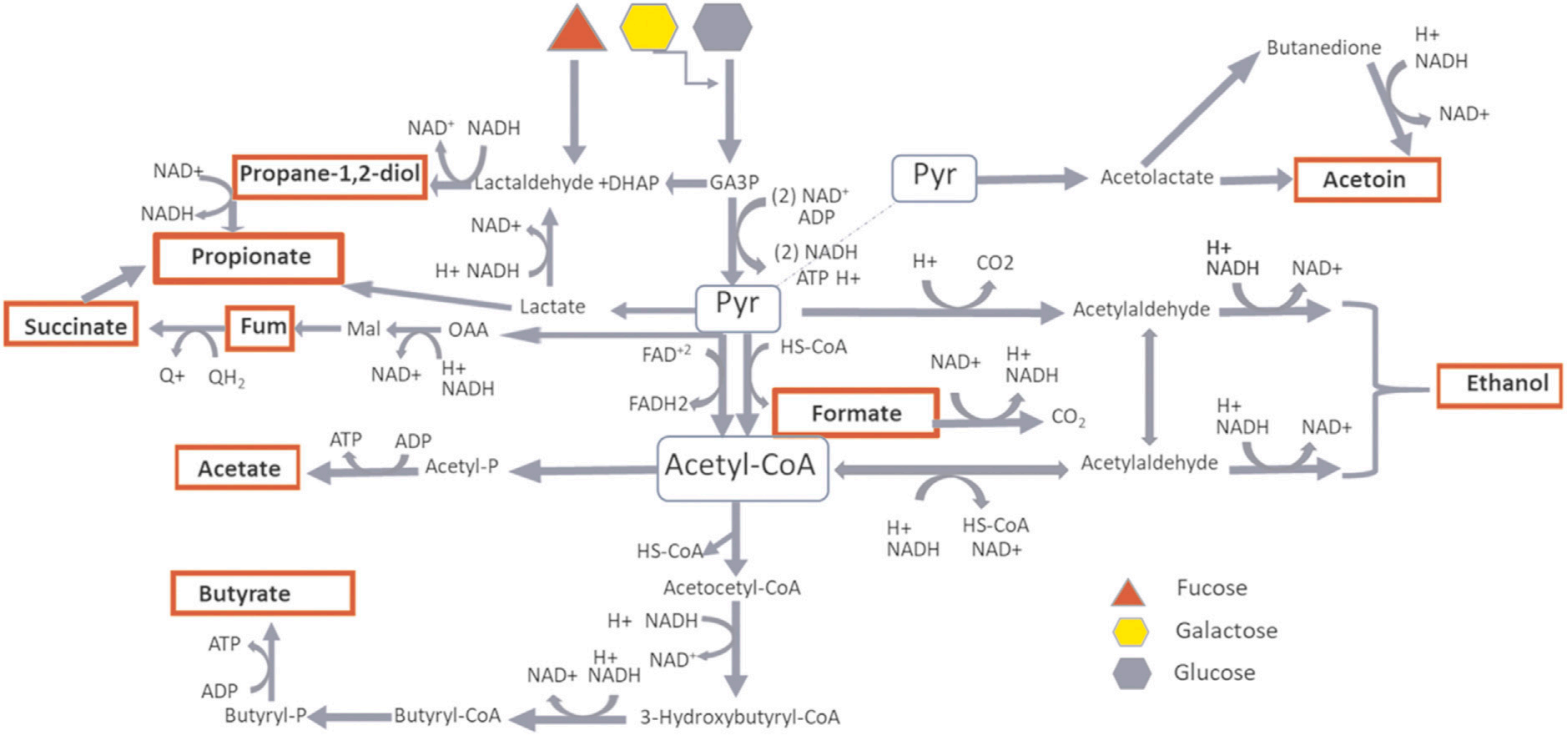
Metabolic pathway of carbohydrate fermentation. In red are highlighted the microbial fermentation’s products as measured by NMR spectroscopy. The products depending on Pyruvate hub are predominant during BM and FBM periods, the products depending on AcetylCoA hub are predominant during W period.

In an anaerobic environment, bacteria regulate energy production through the selection of a range of end-products, during the substrate catabolism. This occurs through oxidation/reduction processes which keep the redox balance constant by the control of intracellular pH and ionic strength, crucial factors for the metabolism thermodynamics. It was possible to distinguish the infant’s gut microbiota metabolism between two phases: a first phase mainly dependent on carbohydrate metabolism with lactate, acetate, propionate, succinate and formate as microbial fermentation’s end-products, with pyruvate as metabolic node and a second phase mainly dependent on mixed substrates catabolism producing acetyl-CoA, acetate, ethanol and butyrate as end-products (Figure 10). This arrangement seemed to be in agreement with previous metabolic models describing the microbial metabolic modulation by changes in the redox potential, in a multi-substrate environment (Villano et al., 2017).

## 5 Conclusion

The transition from an exclusive breastfeeding to the introduction of complementary foods in the infant’s diet could be the driving force underlying changes in fecal microbiota metabolism. However, a direct impact of the microbiota composition was not yet observed. The PCA scores plot and the ComDim scores plot turned out to be very similar, thus suggesting that the weight of the metabolome block was higher in determining the variations induced by diet changes than the taxonomical metagenomic one. The values of ComDim salience provided additional confirmation of this observation. Moreover, the exploration of abundances of the main genera presented in stool samples revealed a high variability that appeared not to be dependent on the diet. However, the results were similar enough within each block of days, thus confirming the good reproducibility of a sampling carried out on consecutive days.

Our approach on a single mother-infant dyad could be considered a strong limitation whether we look for a standard physiological reference model. It is well known that the infant gut microbiota is extremely variable among individuals in the first 2 years of life. The aim of our study was to get more information regarding the relationship among microbiota structure, microbiota metabolic functions and diet, and we believe that the proposed model of single dyad can be better explanatory. In general, this information was obtained by using an *in vitro* intestinal model or gut-on-a-chip, simulating the intestinal conditions and the digestion (Ashammakhi et al., 2020). As a consequence, our approach can be able to give more consistent evidence on the relationship between gut microbiota structure and metabolism.

Furthermore, our aim was also to define a protocol study for the microbiota changes during the diet transition from breastfeeding to weaning, to be applied to more other different mother-infant dyads.

The repetition of the sampling for some days during the different periods gave evidence of the sampling reproducibility within consecutive blocks of days; whereas it showed the instability and the fluctuation of microbiota structure as weaning proceeded.

The intestinal ecosystem in the first 10 months of life is yet not stable. Although the gut microbiota composition is strongly influenced by diet transition, the bacterial communities were still in a constant evolution as a function of the infant’s development and intestinal physical-chemical environment. In this phase and during the weaning, in the absence of pathological events, the microbiota metabolism seems to be more important than the single bacterial genomes. Species can be substituted by other species able to ferment those substrates and, therefore, the gut environmental physical-chemical variables (pH, external electron acceptors) appear to be the driving factors conditioning the microbial complex metabolic response.

In this stage of life, the fecal metabolome appeared to be more representative of the diet changes than gut microbiota.

## Data Availability

The original contributions presented in the study are publicly available. This data can be found in NCBI database: PRJNA719939 (https://www.ncbi.nlm.nih.gov/bioproject).

## Abbreviations

1,2-PD, 1,2-propanediol; 2′FL, 2′fucosyllactose; 3′FL, 3′fucosyllactose; 4-HPA, 4-hydroxyphenylacetate; α-Gal, α-galactose; AUROC, area under the receiver operating characteristic curve; CCR, correct classification rate; CCSWA, common components and specific weight analysis; DCV, double cross validation; DMA, dimethylamine; Fuc, fucose; FUT2, fucosyltranferase 2; GlcNAc, N-acetylglucosamine; HMOs, human milk oligosaccharides; HSQC, heteronuclear single quantum correlation; LDFT, lactodifucotetraose; LNDFH I, II, lacto-N-difucohesaose I, II; LNFP I, III, lacto-N-fucopentaose I, III; MB-PLSDA, multi-block partial least squares discriminant analysis; NMC, number of misclassifications; NMR, nuclear magnetic resonance; OTUs, operational taxonomic units; PCA, principal component analysis; PLS, partial least square; PLS-DA, partial least square-discriminant analysis; rDCV, repeated double cross-validation; RP, rank product; SCFA, short-chain fatty acid
