# Supplementary material for: Longitudinal Multi-Omics Study of a Mother-Infant Dyad from Breastfeeding to Weaning: An Individualized Approach to Understand the Interactions Among Diet, Fecal Metabolome and Microbiota Composition
Source: Front Mol Biosci. 2021 Oct 4;8:688440. doi: 10.3389/fmolb.2021.688440 (PMC8520934; doi:10.3389/fmolb.2021.688440)
Supplement: Supplementary file 1 [file DataSheet1.docx]

Supplementary Material

**Supplementary Table S1.** List of metabolites assigned in stools and in hydro-alcoholic extracts of human milk. The assignment was performed on the basis of 2D-NMR TOCSY, HSQC and HMBC experiments. In bold are highlighted the resonances that we have integrated. M: breast milk metabolites; F: stools metabolites.

| **Name** | **Group** | **Assignment** | **Chemical shift (ppm)** | **Molteplicity** | **^13^C (ppm)** | **Note** |
| --- | --- | --- | --- | --- | --- | --- |
|  |  |  |  |  |  |  |
| Biliary Salt 1 |  | **CH_3_** | **0,67** | bs |  | F |
|  |  |  |  |  |  |  |
| Biliary Salt 2 |  | **CH_3_** | **0,73** | bs |  | F |
|  |  |  |  |  |  |  |
| Ala |  | CH | 3,78 | q |  | M, F |
|  |  | **CH_3_** | **1,48** | d |  |  |
|  |  |  |  |  |  |  |
| Val |  | α-CH | 3,61 | d |  | M, F |
|  |  | β-CH | 2,25 | m |  |  |
|  |  | γ-CH_3_ | 1,05 | d |  |  |
|  |  | **CH_3_** | **0,99** | d |  |  |
|  |  |  |  |  |  |  |
| Leu |  | α-CH | 3,70 | t |  | M, F |
|  |  | β-CH_2_, γ-CH | 1,71 | m |  |  |
|  |  | δ,δ'-CH_3_ | 0,96 | dd |  |  |
|  |  |  |  |  |  |  |
| Ile |  | α-CH | 3,66 |  |  | F |
|  |  | β-CH | 1,96 |  |  |  |
|  |  | ϒ'-CH | 1,46 |  |  |  |
|  |  | ϒ-CH | 1,23 |  |  |  |
|  |  | **CH_3_** | **1,01** | d |  |  |
|  |  |  |  |  |  |  |
| Thr |  | β-CH | 4,27 | m |  | M, F |
|  |  | α-CH | 3,59 | d |  |  |
|  |  | **CH_3_** | **1,33** | d |  |  |
|  |  |  |  |  |  |  |
| Glu |  | α-CH | 3,78 |  |  | M, F |
|  |  | **ϒ-CH_2_** | **2,36** | pt | 36 |  |
|  |  | β-CH_2_ | 2,09 | m |  |  |
|  |  |  |  |  |  |  |
| Gln |  | α-CH | 3,78 |  |  | M, F |
|  |  | **ϒ-CH_2_** | **2,46** | m |  |  |
|  |  | β-CH_2_ | 2,12 | m |  |  |
|  |  |  |  |  |  |  |
| Lys |  | α-CH | 3,75 |  |  | F |
|  |  | ε-CH_2_ | 3,03 | t |  |  |
|  |  | δ-CH_2_ | 1,71 |  |  |  |
|  |  | ϒ-CH_2_ | 1,48 |  |  |  |
|  |  |  |  |  |  |  |
| Phe |  | **5H ring** | **7,23-7,46** | m | 131,5 | F |
|  |  |  |  |  |  |  |
| Tyr |  | CH-2,6 | 7,20 | m |  | F |
|  |  | **CH-3,5** | **6,90** | pd | 119 |  |
|  |  |  |  |  |  |  |
| Asn |  | α-CH | 4,20 |  |  | F |
|  |  | β'-CH | 2,99 | dd |  |  |
|  |  | β-CH | 2,82 | dd |  |  |
|  |  |  |  |  |  |  |
| Gly |  | CH_2_ | 3,56 | s |  | F |
|  |  |  |  |  |  |  |
| His |  | CH-5 ring | 7,86 | s |  | F |
|  |  | **CH-2 ring** | **7,09** | s |  |  |
|  |  |  |  |  |  |  |
| Pro |  | α-CH | 4,13 | t |  | F |
|  |  | β-CH | 2,35 | m |  |  |
|  |  | β'-CH | 2,02 |  |  |  |
|  |  |  |  |  |  |  |
| β-Ala |  | β-CH_2_ | 3,18 | t |  | F |
|  |  | **α-CH_2_** | **2,56** | t |  |  |
|  |  |  |  |  |  |  |
| 1-MeHis |  | CH-5 ring | 7,86 | s |  | F |
|  |  | **CH-2 ring** | **7,09** | s |  |  |
|  |  | CH_3_ | 3,16 | s |  |  |
|  |  |  |  |  |  |  |
| Lactate |  | CH | 4,12 | q |  | M, F |
|  |  | **CH_3_** | **1,33** | d |  |  |
|  |  |  |  |  |  |  |
| Propionate |  | **CH_3_** | **1,06** | t |  | F |
|  |  | CH_2_ | 2,18 | q |  |  |
|  |  |  |  |  |  |  |
| Butyrate |  | α-CH_2_ | 2,16 | t |  | M, F |
|  |  | β-CH_2_ | 1,56 | m |  |  |
|  |  | **γ-CH_3_** | **0,90** | t |  |  |
|  |  |  |  |  |  |  |
| Succinate |  | **α,β-CH_2_** | **2,41** | s | 37 | F |
|  |  |  |  |  |  |  |
| Acetate |  | **CH_3_** | **1,92** | s | 26 | M, F |
|  |  |  |  |  |  |  |
| Piruvate |  | **CH_3_** | **2,37** | s |  | F |
|  |  |  |  |  |  |  |
| Citrate |  | α',γ'-CH | 2,70 | d |  | M |
|  |  | **α,γ-CH** | **2,54** | d |  |  |
|  |  |  |  |  |  |  |
| Acetoine |  | CH | 4,42 | q |  | F |
|  |  | CH_3_ | 2,23 | s |  |  |
|  |  | **CH_3_** | **1,38** | d |  |  |
|  |  |  |  |  |  |  |
| 1,2-Propanediol |  | CH | 3,87 |  |  | F |
|  |  | CH_2_ | 3,50 |  |  |  |
|  |  | **CH_3_** | **1,13** | d |  |  |
|  |  |  |  |  |  |  |
| Methanol |  | **CH_3_** | **3,36** | s | 52 | M,F |
|  |  |  |  |  |  |  |
| Ethanol |  | CH_2_ | 3,65 | q |  | F |
|  |  | **CH_3_** | **1,18** | t |  |  |
|  |  |  |  |  |  |  |
| N-Acetyl |  | **CH_3_** | **2,02 - 2,08** | s | 25 | M, F |
|  |  |  |  |  |  |  |
| 2-Oxoglutarate |  | β-CH_2_ | 3,01 | t |  | M, F |
|  |  | ϒ-CH_2_ | 2,44 | t |  |  |
|  |  |  |  |  |  |  |
| DMA |  | **CH_3_** | **2,74** | s |  | M |
|  |  |  |  |  |  |  |
| TMA |  | **CH_3_** | **2,90** | s |  | M, F |
|  |  |  |  |  |  |  |
| Creatine |  | CH_2_ | 3,90 | s | 56,7 | M, F |
|  |  | **CH_3_** | **3,04** | s | 39 |  |
|  |  |  |  |  |  |  |
| Creatinine |  | CH_2_ | 4,04 | s |  | M |
|  |  | **CH_3_** | **3,05** | s |  |  |
|  |  |  |  |  |  |  |
| Choline |  | CH_2_ | 4,06 |  | 75 | M, F |
|  |  | CH_2_ | 3,55 |  |  |  |
|  |  | **N(CH_3_)_3_** | **3,21** | s | 57 |  |
|  |  |  |  |  |  |  |
| GPC |  | CH_2_ | 4,32 |  |  | M |
|  |  | **N(CH_3_)_3_** | **3,23** | s |  |  |
|  |  |  |  |  |  |  |
| Malonate |  | **CH_2_** | **3,13** | s | 55 | M, F |
|  |  |  |  |  |  |  |
| Taurine |  | CH_2_ | 3,43 | t |  | M, F |
|  |  | CH_2_ | 3,26 | t |  |  |
|  |  |  |  |  |  |  |
| 2-Methylbutyrate |  | α-CH | 2,18 |  |  | F |
|  |  | β-CH_2_ | 1,42 |  |  |  |
|  |  | CH_3_ | 1,05 | d |  |  |
|  |  | **γ-CH_3_** | **0,86** | t |  |  |
|  |  |  |  |  |  |  |
| Methylamine |  | **CH_3_** | **2,60** | s | 27 | F |
|  |  |  |  |  |  |  |
| 2-Aminoisobutyrate |  | CH_3_, CH_3_’ | 1,47 | bs | 19 | F |
|  |  |  |  |  |  |  |
| Methionine |  | **β-CH_2_** | **2,64** | t |  | F |
|  |  | γ-CH_2_ | 2,13 | t |  |  |
|  |  |  |  |  |  |  |
| Fumarate |  | **α,β-CH** | **6,52** | s |  | F |
|  |  |  |  |  |  |  |
| Formate |  | **CH** | **8,46** | s |  | F |
|  |  |  |  |  |  |  |
| Myo-Insositol |  | **CH-1** | **4,06** | pt |  | M, F |
|  |  | CH-2,6 | 3,63 |  |  |  |
|  |  | CH-4 | 3,25 |  | 75 |  |
|  |  |  |  |  |  |  |
| Uracil |  | CH-1 | 7,54 | d |  | F |
|  |  | CH-2 | 5,81 | d | 44,5 |  |
|  |  |  |  |  |  |  |
| Nicotinamide |  | **CH-1** | **8,93** | bs |  | F |
|  |  | CH-5 | 8,61 | bd |  |  |
|  |  | CH-3 | 8,22 | bd |  |  |
|  |  | CH-4 | 7,52 | bd |  |  |
|  |  |  |  |  |  |  |
| 4*-*Hydroxyphenylacetate |  | CH-2,6 | 7,18 |  |  | F |
|  |  | **CH-3,5** | **6,85** | pd | 118 |  |
|  |  |  |  |  |  |  |
| Lactose | Gal (β 1-4) | CH-1 | 4,46 | d |  | M |
|  |  | CH-4 | 3,94 |  |  |  |
|  |  | CH-3 | 3,68 |  |  |  |
|  |  | CH-2 | 3,56 |  |  |  |
|  | α-Glc | **CH-1** | **5,23** | d |  |  |
|  |  | CH-4 | 3,97 |  |  |  |
|  |  | CH-6 | 3,85 |  |  |  |
|  |  | CH-3 | 3,68 |  |  |  |
|  |  | CH-2 | 3,59 |  |  |  |
|  | β-Glc | CH-1 | 4,67 | d |  |  |
|  |  | CH-4 | 3,97 |  |  |  |
|  |  | CH-6 | 3,79 |  |  |  |
|  |  | CH-3 | 3,65 |  |  |  |
|  |  | CH-2 | 3,29 |  |  |  |
|  |  |  |  |  |  |  |
| 3'-SL | Neu5Ac(α2-3) | CH-5 | 3,84 |  |  | M, F |
|  |  | CH-6 | 3,66 |  |  |  |
|  |  | CH-3 | 2,76 | m | 42,7 |  |
|  |  | **CH-3'** | **1,80** | m | 42,7 |  |
|  |  |  |  |  |  |  |
| 6'-SL | Neu5Ac(α2-6) | CH-5 | 3,84 |  |  | M, F |
|  |  | CH-6 | 3,66 |  |  |  |
|  |  | **CH-3** | **2,67** | m | 42,7 |  |
|  |  | CH-3' | 1,73 | m | 42,7 |  |
|  |  |  |  |  |  |  |
| 3’-FL | Fuc(α1-3)βGlc | **CH-1** | **5,38** | d | 101,6 | M , F |
|  |  | CH-3 | 3,96 |  | 18 |  |
|  |  | CH-4 | 3,81 |  |  |  |
|  |  | CH3 | 1,19 |  |  |  |
|  | Fuc(α1-3)αGlc | **CH-1** | **5,43** | d | 101,1 |  |
|  |  | CH-3 | 3,96 |  |  |  |
|  |  | CH-4 | 3,81 |  |  |  |
|  |  | CH3 | 1,19 |  |  |  |
|  |  |  |  |  |  |  |
| LNFP III | Fuc(α1-3)GlcNAc | **CH-1** | **5,13** | d | 101,6 | M, F |
|  |  | **CH-1** | **5,11** | d | 101,6 |  |
|  |  | CH-5 | 4,83 |  | 69 |  |
|  |  | CH-4 | 3,81 |  |  |  |
|  |  | CH3 | 1,19 |  | 18 |  |
|  |  |  |  |  |  |  |
| 2'FL | Fuc(α1-2) | **CH-1** | **5,32** | d | 102,3 | M, F |
|  |  | CH-5 | 4,24 | dd | 69 |  |
|  |  | CH-4 | 3,81 |  |  |  |
|  |  | CH3 | 1,23 | d | 18,34 |  |
|  |  |  |  |  |  |  |
| LNFP I | [Fuc(α1-2)] | **CH-1** | **5,32** | d | 102,3 | M, F |
|  |  | CH-3 | 4,02 |  |  |  |
|  |  | CH3 | 1,24 |  |  |  |
|  |  |  |  |  |  |  |
| Galactose moiety | Gal(β1-4)GlcNac | **CH-1** | **4,43** | d | 104,9 | M, F |
|  |  | CH-4 | 4,14 |  |  |  |
|  |  | CH-6 | 3,90 |  |  |  |
|  |  | CH-6’ | 3,67 |  |  |  |
|  |  | CH-5 | 3,50 |  |  |  |
|  |  |  |  |  |  |  |
| LNDFH I | Fuc(α1-2) | CH-1 | 5,16 | d | 102,6 | M, F |
|  |  | **CH-5** | **4,34** | d | 69 |  |
|  |  | CH-4 | 3,75 |  |  |  |
|  |  | CH3 | 1,27 |  | 18 |  |
|  |  |  |  |  |  |  |
| LNDFH II | Fuc(α1-3)αGlc | CH-1 | 5,38 |  | 102 | M, F |
|  |  | CH-4 | 3,79 |  |  |  |
|  | Fuc(α1-3)βGlc | CH-1 | 5,43 |  | 101,7 |  |
|  |  | CH-4 | 3,79 |  |  |  |
|  |  |  |  |  |  |  |
| Lactosyl moiety | αGlc | **CH-1** | **5,23** | pt | 95 | M, F |
|  |  | CH-6 | 3,87 |  |  |  |
|  |  | CH-1 | 5,19 |  | 95 |  |
|  | Gal(β1-4) | CH-6 | 3,69 |  |  |  |
|  |  | CH-2 | 3,60 |  |  |  |
|  | βGlc | CH-2 | 3,40 |  |  |  |
|  |  |  |  |  |  |  |
| Fucose moiety | Fuc(α1-3)GlcNAc | **CH-1** | **5,19** | d | 103 | M, F |
|  |  | CH-4 | 3,95 |  |  |  |
|  |  | CH-3 | 3,75 |  |  |  |
|  |  | CH3 | 1,20 |  |  |  |
|  |  |  |  |  |  |  |
| LDFT | Fuc(α1-2) | **CH-1** | **5,29** | d | 102,4 | M, F |
|  |  | CH-5 | 4,24 | dd |  |  |
|  |  | CH-4 | 3,81 |  |  |  |
|  |  | CH3 | 1,28 |  |  |  |
|  |  |  |  |  |  |  |
| β-Arabinose |  | **CH-1** | **4,53** | d | 103 | M, F |
|  |  | CH-4,5 | 3,91 |  |  |  |
|  |  | CH-3,5 | 3,66 |  |  |  |
|  |  |  |  |  |  |  |
| Xylose |  | **CH-1** | **4,58** | d | 99,5 | M, F |
|  |  | CH-5 | 3,93 |  |  |  |
|  |  | CH-3 | 3,63 |  |  |  |
|  |  |  |  |  |  |  |
| α-Galactose |  | **CH-1** | **5,27** | d | 95 | F |
|  |  | CH-4 | 3,98 |  |  |  |
|  |  | CH-3 | 3,81 |  |  |  |
|  |  |  |  |  |  |  |
| β-Glucose |  | **CH-1** | **4,65** |  |  | M, F |
|  |  | CH-6 | 3,88 |  |  |  |
|  |  | CH-6 | 3,71 |  |  |  |
|  |  | CH-5 | 3,44 |  |  |  |
|  |  | CH-2 | 3,21 |  |  |  |
|  |  |  |  |  |  |  |
| U01 |  |  | 0,96 | d |  | F |
|  |  | **CH_3_** | **0,79** | d |  |  |
|  |  |  |  |  |  |  |
| U02 |  |  | 2,01 |  |  | F |
|  |  |  | 0,97 | d |  |  |
|  |  | **CH_3_** | **0,83** | d |  |  |
|  |  |  |  |  |  |  |
| U03 |  | **CH_2_** | **4,39** | d |  |  |
|  |  |  | 2,83 |  |  |  |
|  |  |  | 2,68 |  |  | F |
|  |  |  |  |  |  |  |
| U04 |  | **CH_2_** | **4,49** | d |  | F |
|  |  |  | 3,94 |  |  |  |
|  |  |  | 3,68 |  |  |  |
|  |  |  | 3,53 |  |  |  |
|  |  |  |  |  |  |  |
| U 05 |  | **CH** | **7,04** | t |  | F |
|  |  |  | 6,99 | dd |  |  |
|  |  |  |  |  |  |  |
| U06 |  | **CH** | **8,43** | bs |  | F |
|  |  |  |  |  |  |  |
| U07 |  | **CH** | **8,55** | bs |  | F |
|  |  |  |  |  |  |  |
| U08 |  | **CH** | **7,97** | d |  | M |
|  |  |  | 6,13 | d |  |  |

List of abbreviations: Glc Glucose; Gal Galactose; Fuc Fucose; Neu5Ac N-acetylneuraminic acid; 2’FL 2’ fucosyllactose; 3'FL 3' fucosyllactose; 3’SL 3’sialyllactose; 6’SL 6’sialyllactose; LDFT lactodifucotetraose; LNDFH I, II lacto-N-difucohesaose I, II; LNFP I,III lacto-N-fucopentaose I, III; GlcNAc N-acetylglucosamine.

**Supplementary Table S2.** OTUs at level 5 (genus) included in the analysis, based on their detectabilty ( > 80% per sample).

| k__Archaea;p__Euryarchaeota;c__Methanobacteria;o__Methanobacteriales;f__Methanobacteriaceae;g__Methanobrevibacter | | | | | | | | | | |
| --- | --- | --- | --- | --- | --- | --- | --- | --- | --- | --- |
| k__Bacteria;p__Actinobacteria;c__Actinobacteria;o__Actinomycetales;f__Actinomycetaceae;g__Actinomyces | | | | | | | | | |  |
| k__Bacteria;p__Actinobacteria;c__Actinobacteria;o__Bifidobacteriales;f__Bifidobacteriaceae;g__Bifidobacterium | | | | | | | | | |  |
| k__Bacteria;p__Actinobacteria;c__Coriobacteriia;o__Coriobacteriales;f__Coriobacteriaceae;g__Collinsella | | | | | | | | | |  |
| k__Bacteria;p__Actinobacteria;c__Coriobacteriia;o__Coriobacteriales;f__Coriobacteriaceae;g__Eggerthella | | | | | | | | | |  |
| k__Bacteria;p__Bacteroidetes;c__Bacteroidia;o__Bacteroidales;f__Bacteroidaceae;g__Bacteroides |  |  |  |  |  |  |  |  |  |  |
| k__Bacteria;p__Bacteroidetes;c__Bacteroidia;o__Bacteroidales;f__Prevotellaceae;g__Prevotella | | | | | | | | |  |  |
| k__Bacteria;p__Bacteroidetes;c__Bacteroidia;o__Bacteroidales;f__Rikenellaceae;g__ | | | | | | | |  |  |  |
| k__Bacteria;p__Bacteroidetes;c__Bacteroidia;o__Bacteroidales;f__[Barnesiellaceae];g__ | | | | | | | |  |  |  |
| k__Bacteria;p__Bacteroidetes;c__Flavobacteriia;o__Flavobacteriales;f__Cryomorphaceae;g__Fluviicola | | | | | | | | |  |  |
| k__Bacteria;p__Bacteroidetes;c__Flavobacteriia;o__Flavobacteriales;f__Flavobacteriaceae;g__Flavobacterium | | | | | | | | | |  |
| k__Bacteria;p__Cyanobacteria;c__Chloroplast;o__Streptophyta;f__;g__ | | | | | |  |  |  |  |  |
| k__Bacteria;p__Firmicutes;c__Bacilli;o__Bacillales;f__Planococcaceae;g__ | | | | | | |  |  |  |  |
| k__Bacteria;p__Firmicutes;c__Bacilli;o__Bacillales;f__Staphylococcaceae;g__Staphylococcus | | | | | | | |  |  |  |
| k__Bacteria;p__Firmicutes;c__Bacilli;o__Gemellales;f__Gemellaceae;g__ | | | | | |  |  |  |  |  |
| k__Bacteria;p__Firmicutes;c__Bacilli;o__Lactobacillales;f__Carnobacteriaceae;g__Granulicatella | | | | | | | | |  |  |
| k__Bacteria;p__Firmicutes;c__Bacilli;o__Lactobacillales;f__Enterococcaceae;g__Enterococcus |  |  |  |  |  |  |  |  |  |  |
| k__Bacteria;p__Firmicutes;c__Bacilli;o__Lactobacillales;f__Lactobacillaceae;g__Lactobacillus |  |  |  |  |  |  |  |  |  |  |
| k__Bacteria;p__Firmicutes;c__Bacilli;o__Lactobacillales;f__Streptococcaceae;g__Streptococcus |  |  |  |  |  |  |  |  |  |  |
| k__Bacteria;p__Firmicutes;c__Bacilli;o__Turicibacterales;f__Turicibacteraceae;g__Turicibacter | | | | | | | | |  |  |
| k__Bacteria;p__Firmicutes;c__Clostridia;o__Clostridiales;f__;g__ | | | | | |  |  |  |  |  |
| k__Bacteria;p__Firmicutes;c__Clostridia;o__Clostridiales;f__Clostridiaceae;g__ |  |  |  |  |  |  |  |  |  |  |
| k__Bacteria;p__Firmicutes;c__Clostridia;o__Clostridiales;f__Clostridiaceae;g__Clostridium |  |  |  |  |  |  |  |  |  |  |
| k__Bacteria;p__Firmicutes;c__Clostridia;o__Clostridiales;f__Clostridiaceae;g__SMB53 |  |  |  |  |  |  |  |  |  |  |
| k__Bacteria;p__Firmicutes;c__Clostridia;o__Clostridiales;f__Lachnospiraceae;g__ |  |  |  |  |  |  |  |  |  |  |
| k__Bacteria;p__Firmicutes;c__Clostridia;o__Clostridiales;f__Lachnospiraceae;g__Anaerostipes | | | | | | | | |  |  |
| k__Bacteria;p__Firmicutes;c__Clostridia;o__Clostridiales;f__Lachnospiraceae;g__Blautia | | | | | | | |  |  |  |
| k__Bacteria;p__Firmicutes;c__Clostridia;o__Clostridiales;f__Lachnospiraceae;g__Coprococcus | | | | | | | |  |  |  |
| k__Bacteria;p__Firmicutes;c__Clostridia;o__Clostridiales;f__Lachnospiraceae;g__Dorea | | | | | | | |  |  |  |
| k__Bacteria;p__Firmicutes;c__Clostridia;o__Clostridiales;f__Lachnospiraceae;g__Epulopiscium | | | | | | | | |  |  |
| k__Bacteria;p__Firmicutes;c__Clostridia;o__Clostridiales;f__Lachnospiraceae;g__[Ruminococcus] | | | | | | | | |  |  |
| k__Bacteria;p__Firmicutes;c__Clostridia;o__Clostridiales;f__Peptostreptococcaceae;g__ | | | | | | | |  |  |  |
| k__Bacteria;p__Firmicutes;c__Clostridia;o__Clostridiales;f__Ruminococcaceae;g__ | | | | | | |  |  |  |  |
| k__Bacteria;p__Firmicutes;c__Clostridia;o__Clostridiales;f__Ruminococcaceae;g__Faecalibacterium | | | | | | | | |  |  |
| k__Bacteria;p__Firmicutes;c__Clostridia;o__Clostridiales;f__Ruminococcaceae;g__Oscillospira | | | | | | | | |  |  |
| k__Bacteria;p__Firmicutes;c__Clostridia;o__Clostridiales;f__Ruminococcaceae;g__Ruminococcus |  |  |  |  |  |  |  |  |  |  |
| k__Bacteria;p__Firmicutes;c__Clostridia;o__Clostridiales;f__Veillonellaceae;g__Dialister | | | | | | | |  |  |  |
| k__Bacteria;p__Firmicutes;c__Clostridia;o__Clostridiales;f__Veillonellaceae;g__Megasphaera | | | | | | | |  |  |  |
| k__Bacteria;p__Firmicutes;c__Clostridia;o__Clostridiales;f__Veillonellaceae;g__Phascolarctobacterium | | | | | | | | |  |  |
| k__Bacteria;p__Firmicutes;c__Clostridia;o__Clostridiales;f__Veillonellaceae;g__Veillonella |  |  |  |  |  |  |  |  |  |  |
| k__Bacteria;p__Firmicutes;c__Clostridia;o__Clostridiales;f__[Mogibacteriaceae];g__ | | | | | | | |  |  |  |
| k__Bacteria;p__Firmicutes;c__Erysipelotrichi;o__Erysipelotrichales;f__Erysipelotrichaceae;g__ | | | | | | | | |  |  |
| k__Bacteria;p__Firmicutes;c__Erysipelotrichi;o__Erysipelotrichales;f__Erysipelotrichaceae;g__[Eubacterium] | | | | | | | | | |  |
| k__Bacteria;p__Fusobacteria;c__Fusobacteriia;o__Fusobacteriales;f__Fusobacteriaceae;g__Fusobacterium | | | | | | | | | |  |
| k__Bacteria;p__Proteobacteria;c__Gammaproteobacteria;o__Aeromonadales;f__Aeromonadaceae;g__ | | | | | | | | |  |  |
| k__Bacteria;p__Proteobacteria;c__Gammaproteobacteria;o__Enterobacteriales;f__Enterobacteriaceae;g__ |  |  |  |  |  |  |  |  |  |  |
| k__Bacteria;p__Proteobacteria;c__Gammaproteobacteria;o__Enterobacteriales;f__Enterobacteriaceae;g__Citrobacter |  |  |  |  |  |  |  |  |  |  |
| k__Bacteria;p__Proteobacteria;c__Gammaproteobacteria;o__Enterobacteriales;f__Enterobacteriaceae;g__Erwinia |  |  |  |  |  |  |  |  |  |  |
| k__Bacteria;p__Proteobacteria;c__Gammaproteobacteria;o__Enterobacteriales;f__Enterobacteriaceae;g__Klebsiella | | | | | | | | | | |
| k__Bacteria;p__Proteobacteria;c__Gammaproteobacteria;o__Enterobacteriales;f__Enterobacteriaceae;g__Serratia | | | | | | | | | |  |
| k__Bacteria;p__Proteobacteria;c__Gammaproteobacteria;o__Enterobacteriales;f__Enterobacteriaceae;g__Trabulsiella | | | | | | | | | | |
| k__Bacteria;p__Proteobacteria;c__Gammaproteobacteria;o__Pasteurellales;f__Pasteurellaceae;g__ | | | | | | | | |  |  |
| k__Bacteria;p__Proteobacteria;c__Gammaproteobacteria;o__Pasteurellales;f__Pasteurellaceae;g__Actinobacillus | | | | | | | | | |  |
| k__Bacteria;p__Proteobacteria;c__Gammaproteobacteria;o__Pasteurellales;f__Pasteurellaceae;g__Haemophilus |  |  |  |  |  |  |  |  |  |  |
| k__Bacteria;p__Proteobacteria;c__Gammaproteobacteria;o__Pseudomonadales;f__Moraxellaceae;g__Acinetobacter | | | | | | | | | | |
| k__Bacteria;p__Proteobacteria;c__Gammaproteobacteria;o__Pseudomonadales;f__Pseudomonadaceae;g__Pseudomonas | | | | | | | | | | |
| k__Bacteria;p__Verrucomicrobia;c__Verrucomicrobiae;o__Verrucomicrobiales;f__Verrucomicrobiaceae;g__Akkermansia | | | | | | | | | | |

**Supplementary Table S3.** Sample collection, diet and related omics analyses performed on each sample.

| **Days after birth** | **Diet** | **Metabolomics** | **Metagenomics** |
| --- | --- | --- | --- |
| 103 | 4 Breast Milk (BM) | yes | yes |
| 104 | 4 BM | yes | yes |
| 105 | 4 BM | yes | yes |
| 106 | 4 BM | yes | yes |
| 107 | 4 BM | yes | - |
| 108 | 4 BM | yes | - |
| 145 | 4 BM | yes | yes |
| 146 | 4 BM | yes | yes |
| 147 | 4 BM | yes | yes |
| 165 | 4 BM+1 fruit snack (pear or apple) | yes | - |
| 167 | 4 BM+1 fruit snack | yes | - |
| 168 | 4 BM+1 fruit snack | yes | - |
| 169 | 4 BM+1 fruit snack | yes | - |
| 171 | 4 BM+1 fruit snack | yes | yes |
| 172 | 4 BM+1 fruit snack | yes | yes |
| 173 | 2 BM+1 fruit snack+ 1 vegetable stock with rice or tapioca cream | yes | - |
| 174 | 2 BM+1 fruit snack+ 1 vegetable stock with rice or tapioca cream + homogenized l meat + parmesan cheese | yes | yes |
| 175 | 2 BM+1 fruit snack+ 1 vegetable stock with rice or tapioca cream + homogenized meat + parmesan cheese | yes | yes |
| 219 | 1 BM + 2 semi-solid meal (containing vegetable, meat, fish or cheese) + fruit snack | yes | - |
| 220 | 1 BM + 2 semi-solid meal+ fruit snack | yes | yes |
| 221 | 1 BM + 2 semi-solid meal+ fruit snack | yes | yes |
| 222 | 1 BM + 2 semi-solid meal+ fruit snack | yes | yes |
| 225 | 1 BM + 2 semi-solid meal+ fruit snack | yes | yes |
| 226 | 1 BM + 2 semi-solid meal+ fruit snack | yes | yes |
| 227 | 1 BM + 2 semi-solid meal+ fruit snack | yes | yes |
| 228 | 1 BM + 2 semi-solid meal+ fruit snack | yes | yes |
| 262 | 1 BM + 2 semi-solid meal+ fruit snack | yes | yes |
| 263 | 1 BM + 2 semi-solid meal+ fruit snack | yes | yes |
| 264 | 1 BM + 2 semi-solid meal (introduced eggs)+ fruit snack | yes | yes |
| 265 | 1 BM + 2 semi-solid meal+ fruit snack | yes | yes |
| 266 | 1 BM + 2 semi-solid meal+ fruit snack | yes | yes |
| 267 | 1 BM + 2 semi-solid meal+ fruit snack | yes | yes |
| 268 | 1 BM + 2 semi-solid meal+ fruit snack | yes | yes |


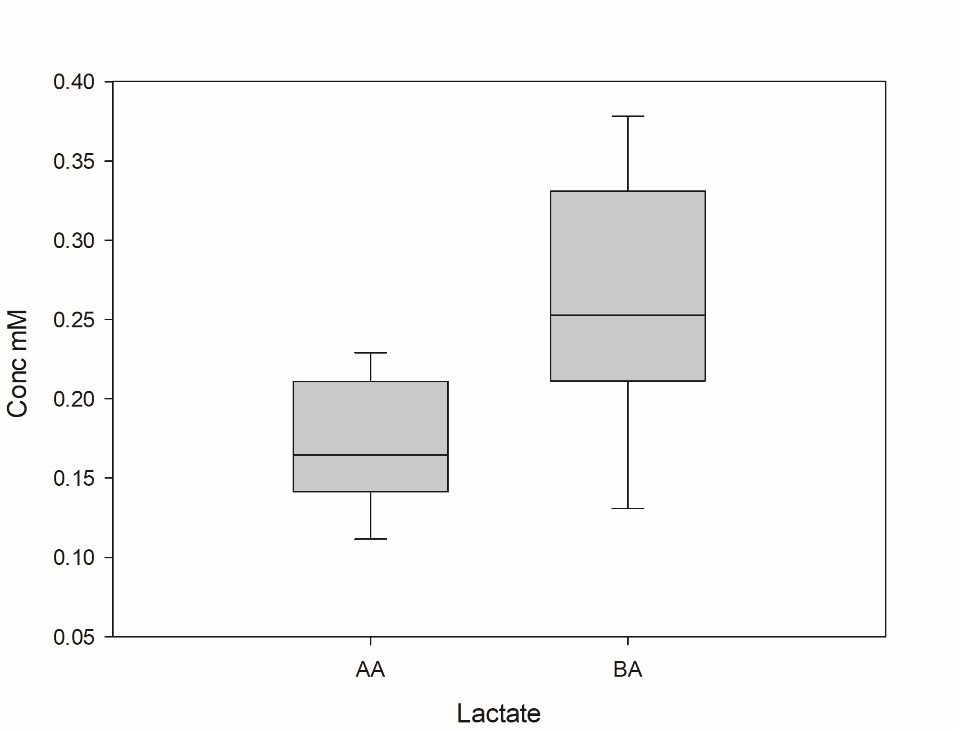


**Supplementary Figure S1.** Two tailed Student’s t-test was applied to assess the differences on the lactate levels at the beginning (AA) and at the end (BA) of the suction. p=0.007.


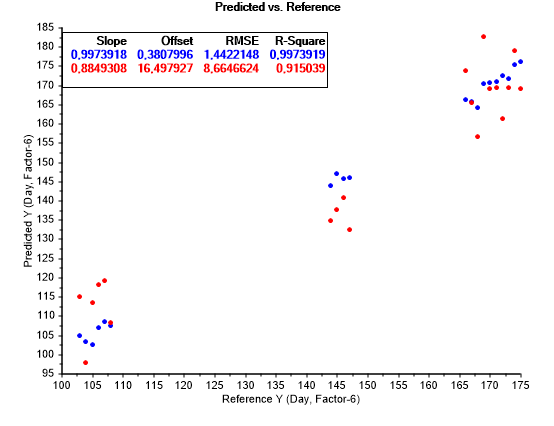

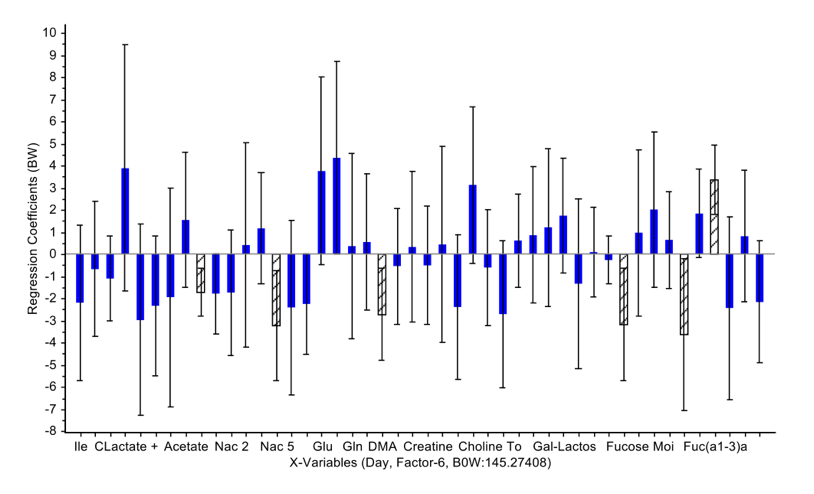


(A) (B)

**Supplementary Figure S2**. (A) R^2^ and Q^2^ and (B) regression coefficients plots of the Partial Least Square (PLS) analysis performed on the breast milk metabolomic matrix to assess the evolution of milk composition as a function of lactation time. The model showed six significant latent variables, with R^2^Y=0.99 and Q^2^Y=0.91. The regression coefficients showed a significant increase (p<0.05) of 3'-fucosyllactose levels and a significant decrease of N-acetyl moieties of oligosaccharides, dimethylamine, lacto-N-fucopentaose III, 2'-fucosyllactose and lacto-N-fucopentaose I levels (dashed bars).

**Supplementary Figure S3.** Histograms with mean and standard deviation for the fecal metabolites at the five considered sampling periods.

**Supplementary Figure S4.** Histograms with mean and standard deviation for the OTUs at the five considered sampling periods.


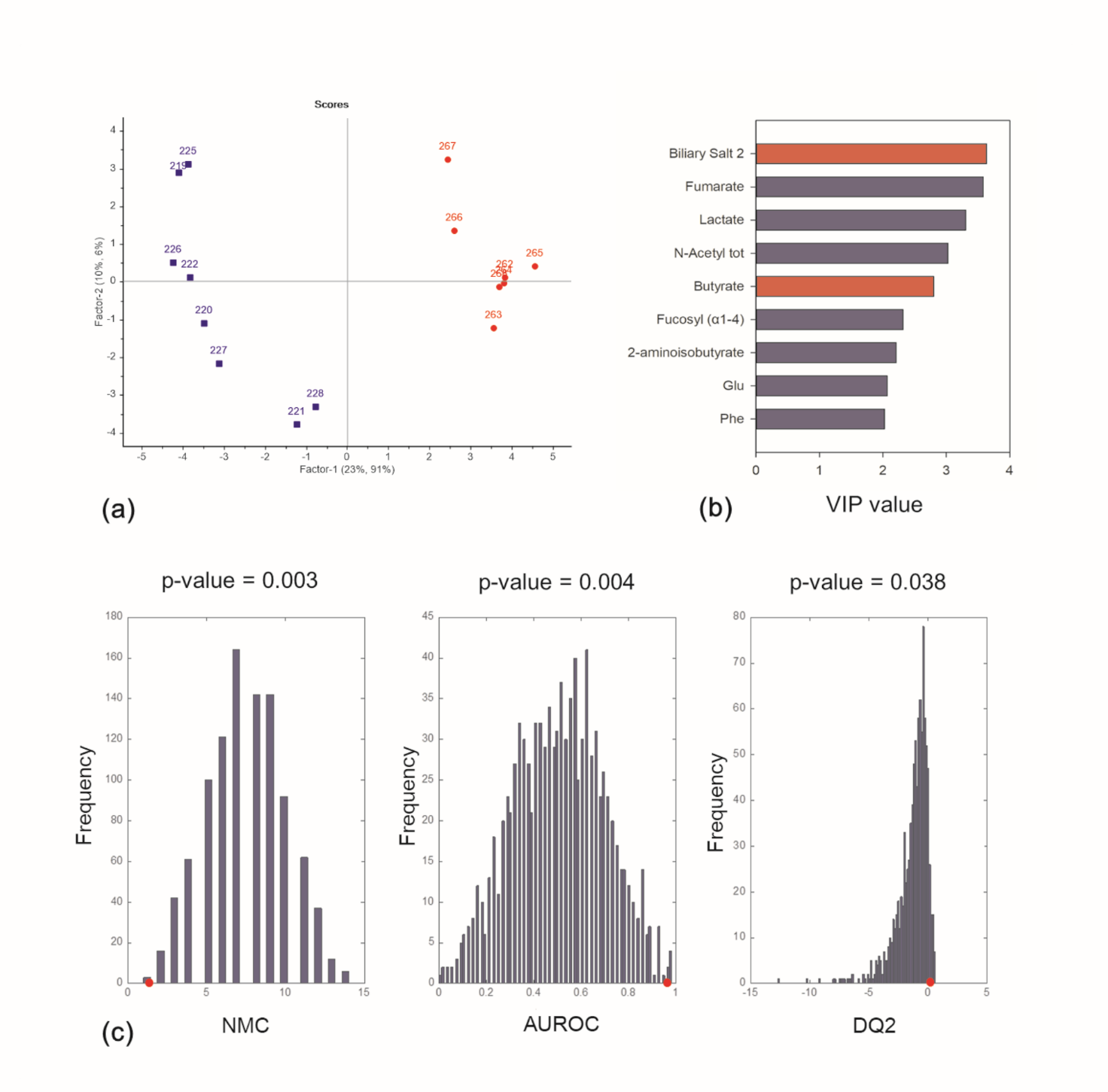


**Supplementary Figure S5**. PLS-DA analysis of fecal waters samples on 219-228 days and 262-268 days.

1. LV scores plot; in blue 219-228 days and in red 262-268 days;
2. VIP scores histograms: only the significant variables (VIP>1.5) were reported.
3. Figures of merit relative to diagnostic statistic tests, namely NMC (Number of Misclassifications), AUROC curve (Area Under the Receiver Operating Characteristic) and DQ^2^ (Discriminant Q^2^).
